# Effects of captopril against radiation injuries in the Göttingen minipig model of hematopoietic-acute radiation syndrome

**DOI:** 10.1371/journal.pone.0256208

**Published:** 2021-08-27

**Authors:** W. Bradley Rittase, Elizabeth A. McCart, Jeannie M. Muir, Roxane M. Bouten, John E. Slaven, Ognoon Mungunsukh, Michelle A. Bylicky, W. Louis Wilkins, Sang-Ho Lee, Kristbjorn O. Gudmundsson, Tiziana Di Pucchio, Cara H. Olsen, Yang Du, Regina M. Day

**Affiliations:** 1 Department of Pharmacology and Molecular Therapeutics, Uniformed Services University of the Health Sciences, Bethesda, MD, United States of America; 2 Department of Pathology, Uniformed Services University of the Health Sciences, Bethesda, MD, United States of America; 3 Division of Comparative Pathology, The Armed Forces Radiobiology Research Institute/Uniformed Services University of Health Sciences, Bethesda, MD, United States of America; 4 Department of Pediatrics, Uniformed Services University of the Health Sciences, Bethesda, MD, United States of America; 5 Department of Preventive Medicine and Biostatistics, Uniformed Services University of the Health Sciences, Bethesda, MD, United States of America; Max Delbruck Centrum fur Molekulare Medizin Berlin Buch, GERMANY

## Abstract

Our laboratory has demonstrated that captopril, an angiotensin converting enzyme inhibitor, mitigates hematopoietic injury following total body irradiation in mice. Improved survival in mice is correlated with improved recovery of mature blood cells and bone marrow, reduction of radiation-induced inflammation, and suppression of radiation coagulopathy. Here we investigated the effects of captopril treatment against radiation injuries in the Göttingen mini pig model of Hematopoietic-Acute Radiation Syndrome (H-ARS). Minipigs were given captopril orally (0.96 mg/kg) twice daily for 12 days following total body irradiation (^60^Co 1.79 Gy, 0.42–0.48 Gy/min). Blood was drawn over a time course following irradiation, and tissue samples were collected at euthanasia (32–35 days post-irradiation). We observed improved survival with captopril treatment, with survival rates of 62.5% in vehicle treated and 87.5% in captopril treated group. Additionally, captopril significantly improved recovery of peripheral blood mononuclear cells, and a trend toward improvement in recovery of red blood cells and platelets. Captopril significantly reduced radiation-induced expression of cytokines erythropoietin and granulocyte-macrophage colony-stimulating factor and suppressed radiation-induced acute-phase inflammatory response cytokine serum amyloid protein A. Using quantitative-RT-PCR to monitor bone marrow recovery, we observed significant suppression of radiation-induced expression of redox stress genes and improved hematopoietic cytokine expression. Our findings suggest that captopril activities in the Göttingen minipig model of hematopoietic-acute radiation syndrome reflect findings in the murine model.

## Introduction

Despite the end of the cold war, there still exists a nuclear threat from a terrorist attack. Additionally, increases in the use of nuclear technology and nuclear energy are associated with increased risk of toxic accidental radiation exposure [1, 2]. Exposure to total body irradiation can result in acute toxicities to multiple organs, collectively known as Acute Radiation Syndrome (ARS). Several treatments have been approved by the US Food and Drug Administration (FDA) for the treatment of hematopoietic subsyndrome of ARS (H-ARS), including two preparations of granulocyte colony-stimulating factor (G-CSF), one preparation of granulocyte-macrophage colony-stimulating factor (GM-CSF), and one preparation of romiplostim, a thrombopoietin receptor agonist [1, 3]. However, G-CSF, GM-CSF, and romiplostim are injectable treatments most often administered under medical supervision, and may not be suitable for a mass casualty event. Therefore, research continues toward the development of a safe and effective countermeasure for H-ARS that can be easily administered to large numbers of people.

The development of radiation countermeasures for accidental radiological exposure is governed by the “Animal Rule” of the US FDA [4, 5]. Under the Animal Rule, drugs and biologics can be developed for permit approval or licensing when efficacy studies in humans would be unethical or infeasible [4]. Four primary criteria must be met to provide evidence under the Animal Rule including the demonstration of efficacy in more than one animal species, or in a single species that is predictive for human response [5]. To meet the specific needs for FDA approval of radiation countermeasures, the Göttingen minipig model for H-ARS has been under development [6–11]. These studies have provided measurable dose-response curves of Göttingen minipigs exposed to radiation, the time course of symptoms of radiation exposure, and a comparison of their response to irradiation to other species including humans [7–10, 12]. A study of the response of Göttingen minipigs to G-CSF has also provided evidence that this species can be used to study the efficacy of radiation countermeasures [10].

Captopril is an angiotensin converting enzyme inhibitor that is orally available and has been FDA approved for over three decades for the treatment of hypertension and heart failure [13]. Our laboratory has demonstrated the efficacy of captopril as a countermeasure against H-ARS in a murine model [14–16]. Our studies have shown that in the murine model, captopril can be initiated from 24 h or 48 h post-irradiation for significant improvement in survival [14]. Reduced mortality following total body irradiation in mice by captopril administration is associated with improved mature blood cell recovery, improved bone marrow and hematopoietic progenitor recovery, suppression of radiation-induced acute inflammation, and reduction of radiation coagulopathy [14–17]. Here we have investigated the effects of captopril in the Göttingen minipig model of H-ARS. For this initial study, captopril was administered at 4 h post-irradiation as approved for the funded research.

## Materials and methods

### Reagents and chemicals

Reagents were obtained from Sigma-Aldrich (St. Louis, MO, USA) except where indicated.

### Animals and irradiation

All animal handling procedures were performed in compliance with guidelines from the National Research Council for the ethical handling of laboratory animals and approved by the Uniformed Services University (USU) and Armed Forces Radiobiology Research Institute (AFRRI) Institutional Animal Care and Use Committees. Male Göttingen swine (4–6 months of age, 8–11 kg upon shipment) were obtained from Marshall BioResources of Marshall Farms Group, Ltd. (North Rose, NY, USA). Animals were group housed for communal enrichment in a 20 ± 2°C, 50% ± 10% humidity, and 12:12 light/dark cycle facility accredited by the Association for Assessment and Accreditation of Laboratory Animal Care International. Additional enrichment was provided. Commercial minipig diet (Harlan Teklad Minipig diet 8753, Madison, WI, USA) was provided twice daily. Water was provided *ad libitum*. Animals were weighed and randomized into the following treatment groups: 1) Sham+Vehicle (*n* = 8); 2) Sham+Captopril (*n* = 10); Radiation+Vehicle (*n* = 12); 4) Radiation+ Captopril (*n* = 11). The animal handling staff were not blinded to the treatment groups, but blood analysis staff and veterinary pathologists were blinded to treatment groups. The total animal number for survival was achieved over the course of 3 separate experiments. Swine were acclimatized to the animal facility at USU for 7–10 days prior to the first blood draw at the beginning of the study. In two sets of experiments, temperatures of the animals were taken daily using implantable Programmable Temperature Transponder microchips with a wireless handheld reader (microchip model #IPTT-200, Bio Medic Data Systems, Seaford, DE, USA). To implant microchips 10 days prior to irradiation, skin was shaved and aseptically cleaned. The microchips were injected with disposable needle/syringe/chip assemblies between the subcutaneous fat and muscle layer in the left lateral cervical region, just in front of the cranial margin of the shoulder. Surgical skin glue was used to close the injection site. For other experiments, the temperatures were obtained using a pediatric rectal thermometer, placed with lubrication under anesthesia during blood draws. Approximately two weeks after arrival, swine were given total body irradiation. Animals were injected with telazol/xylazine (4.4mg/kg-2mg/kg) for anesthesia during irradiation. While under anesthesia, swine were positioned in a panepinto sling and exposed bilaterally to a target total body dose of 1.79 Gy of Cobalt (^60^Co) irradiation delivered at a dose rate of 0.485–0.502 Gy/min as previously described [7]. Dosimetry for irradiation was performed according to Moroni et al., 2011 [7], with minor modifications. To determine the dose rates for animals of different widths, dose rates were measured in the cores of cylindrical phantoms of 7.6, 12.7, 17.7 and 20.3 cm, covering the range of animal sizes. Phantoms were filled with water to approximate the tissue-equivalent from the viewpoint of radiation physics of the ^60^Co phantoms. Dose rates were measured before the experiments using alanine pellets, calibrated at the UK National Physical Laboratory in Teddington. Electron paramagnetic resonance (EPR) measurements of alanine dosimeters were done in accordance with the International Organization or Standards/American Society for Testing Materials (ISO/ASTM) [18]. Adherence to this practice yields an estimated expanded uncertainty in absorbed dose within 3%. The target dose was obtained via initial measurement with caliper across the widest midline section, real-time dosimetry with an ionizing chamber (dose rates were previously determined with alanine-EPR system), and calculated with 0.49 Gy/min, total body bilateral exposure. After irradiation, each animal was transported back to the animal facility at USU for recovery. Swine assigned to the sham groups were anesthetized with telazol/Xylazine (4.4 mg/kg-2 mg/kg) in the animal facility. Water was readily available and food was provided twice daily. Antibiotics or additional medical support were not provided under this protocol, so that the effect of captopril administration alone could be tested. Hematopoietic subsyndrome of acute radiation syndrome is considered to occur within 30 days post-irradiation [7]. Therefore, 35 days was selected as the endpoint of the study. Weights and temperatures were taken on the same days as blood draws. Absolute criteria for early euthanasia were non-responsiveness, dypnea, and loss of 20% expected body weight. Non-absolute criteria for early euthanasia were hyper/hypothermia, anorexia (skipping three consecutive BID meals), anemia/pallor, petechiae/ecchymosis, vomiting or diarrhea, lethargy, seizures, ataxia, or vestibular signs, or uncontrollable hemorrhage. Vomiting and diarrhea was not observed in animals following 1.79 Gy total body irradiation. Necropsies were performed on all animals after euthanasia.

### Captopril administration

Captopril (USP grade, Sigma-Aldrich, St Louis, MO, USA), was dissolved in sterile water, made fresh weekly, a preparation previously demonstrated to be stable [19]. Captopril was administered orally, 0.96 mg/kg, twice daily. The dosage of captopril was based on previous studies of captopril effects in Yorkshire or other large pig breeds [20], and adjusted for total body weight. For administration, captopril was mixed in ~1 ml of yogurt immediately before administration. Captopril or vehicle was administered from 4 h through 12 days post-irradiation.

### Tissue, blood, and serum samples

The time points for blood cell, blood chemistries, and blood cytokine analyses were chosen to collect information on blood cell recovery and cytokine changes at least twice weekly, based on findings by Moroni et al., 2011 [7]. The time points for blood cell, blood chemistries, and cytokine analyses were days -5 (pre-irradiation) and days 2, 6, 9, 13, 16, 20., 23, 30, and 35 post-irradiation. We repeated the collection of blood for cytokine analysis one additional time, to obtain EPO levels; in this experiment (5A), blood was collected on day -5 (pre-irradiation, shown as baseline), and days 2, 6, 9, 13, 16, 20, 23, 30, and 35. For hematopoietic progenitor analysis, blood was collected from sham irradiated pigs (basal levels), and from irradiated pigs on days 6, 9, 13, 16, and 23 post-irradiation. Prior to collecting blood samples, minipigs were anesthetized with isoflurane inhalation while secured in a panepinto sling. Blood was collected from the cephalic vein in citrate, SST, and EDTA collection tubes (BD Vacutainer®, Franklin Lakes, NJ, USA). Plasma and serum were isolated, aliquoted and stored at -80°C. Complete blood cell counts (CBC), including reticulocytes, were quantitatively obtained through flow cytometry (ADVIA 2120, Siemens Healthcare Diagnostics, Tarrytown, NY, USA). Blood chemistries were performed on a VITROS 350 Chemistry System (Cardinal Health Instruments, Columbus, OH, USA). Chemistries obtained were alanine aminotransferase (ALT), alkaline phosphatase (ALK/PHOS), creatinine, blood urea nitrogen (BUN), and glucose. To acquire the 35 day post-irradiation blood sample, minipigs were anesthetized using injection of telazol/xylazine followed by cardiocentesis. Animals were then humanely euthanized intravenously with Euthasol®, 4.5 ml/kg (Virbac, Fort Worth, TX, USA) according to current AVMA guidelines. Each animal underwent necropsy for gross pathology and tissue collection. Blood samples that contained clots were not used for blood cell counts or blood chemistries. Histological samples were processed and stained for hematoxylin and Eosin (H&E) and Masson’s Trichrome stain by Histoserve, Inc. (Germantown, MD, USA).

### Colony forming units

Peripheral blood mononuclear cells (PBMCs) were separated from whole blood by density gradient media (Ficoll-Paque™ Plus, Ge Healthcare Bio-Sciences AB, Uppsala, Sweden). Blood cells were washed with Iscove’s Modified Dulbecco’s Medium (IMDM), Glutamax + 2% FBS (#31980048 Gibco, Gaithersburg, MD) and resuspended with ACK lysing buffer to remove remaining RBCs. PBMC pellets were washed with IMDM with 30% FBS, and cells were counted to obtain 2 × 10^6^ cells/dish. PBMCs were plated in duplicates with MethoCult™ H4304 Optimum (StemCell Technologies, Vancouver, Canada). Colony forming units (CFUs) were manually counted 7–14 days later and scored for lineage type.

### Detection of serum serum amyloid A, granulocyte-macrophage colony-stimulating factor, and erythropoietin

Serum Amyloid A (SAA), Swine CSF2 (granulocyte-macrophage colony-stimulating factor, GM-CSF), erythropoietin (EPO) in the serum were detected at time points using Phase™Range Multispecies SAA enzyme-linked immunosorbent assay [ELISA] kit, (Tridelta Development Ltd., Kildare, Ireland), GM-CSF ELISA kit,(Thermo Scientific™, Frederick, MD, USA) and porcine erythropoietin [EPO] ELISA kit (LSBio, Seattle, WA), respectively, and according to manufacturer’s instructions. ELISAs were read in 96 well plates using a BioTek Synergy H1 hybrid plate reader (Winooski, VT, USA).

### RNA isolation, custom gene array, and qRT-PCR

Tissues harvested after euthanasia were immediately placed in RNALater buffer and stored according to the manufacturer’s instructions (Qiagen, Germantown, MD, USA). At the time of RNA purification, tissues were homogenized with an Ultra Turrax homogenizer (Jahnke & Kunkel, Staufen, Germany). Tissue samples were then processed using QIAshredder mini columns (Qiagen) according to the manufacturer’s instructions. RNA was isolated from the homogenate using the RNeasy mini kit (Qiagen) according to the manufacturer’s instructions, and genomic DNA was removed using the RNase-free DNase Set (Qiagen). RNA was quantified spectroscopically (ND-1000 Spectrophotometer, NanoDrop Technologies, Wilmington, DE, USA). 1.0 μg was reverse transcribed using iScript cDNA synthesis kit (Bio-Rad, Hercules, CA, USA), according to the manufacturer’s protocol. Initial screens were performed in triplicate to compare one biological sample from each treatment group (sham irradiation, irradiation + vehicle, and irradiation + captopril), to determine bone marrow gene expression using PrimePCR™ Assays and Controls, using Bio-Rad PrimePCR Assays, using customized and tested gene sequences for *Sus scrofa* and including three reference genes, according to the manufacturer’s protocol. Following initial screens, qPCR was performed using four biological replicates. Complementary DNA (cDNA) was diluted 10-fold with nuclease-free water and 2 μl were used in each 20 μl RT-qPCR reaction. RT-qPCRs were performed with technical duplicates using 300 nM of each primer and 10 μl of iTaq™ Universal SYBR® Green Supermix (Bio-Rad), on a CFX96 Touch Real-Time PCR Detection System (Bio-Rad). Primers for qRT-PCR were designed using NCBI /Primer-BLAST and purchased from Integrated DNA Technologies (Coralville, IA, USA). Sequences for qPCR primers are shown in Table 1. Primers for *Sus scrofa* genes CCL5 (C-C motif chemokine ligand 5), IL5 (interleukin-5), IL10 (interleukin-10), and JAM2 (junctional adhesion molecule 2) were purchased from Bio-Rad Laboratories. Assays were performed according to the manufacturer’s instructions using an iCycler with a IQ5 optical system (Bio-Rad Laboratories). For quantification, the comparative threshold cycle (CT) method was used to assess relative changes in mRNA levels between the untreated control and the drug-treated and/or irradiated animal samples.

**Table 1:** Primers for qPCR.

Gene	Forward Primer	Reverse Primer
CCL2	5’-AGAAGATCTCGATGCAGCGG-3’	5’-TTCTGCTTGGGTTCTGCACA-3’
COL3A1	5’-CCTTGAGGCTGATGGGATCA-3’	5’-TTCGAAGACTGTCTTGCCCC-3’
CXCL8	5’-CGAAGCCAGAACTCTCCCTG-3’	5’-GTTGCATGGCTGTTCACAGG-3’
GAPDH	5’-TCGTTGAAAGGAAGCCCAGA-3’	5’-TCTTCAGTGATGTTGATCAGGAA-3’
IL2	5’-GCTCTGGAGGGAGTGCTAAATT-3’	5’-TGTTCAGAAATTCAACAGCAGTT-3’
IL4	5’-GCTTCGGCACATCTACAGAC-3’	5’GCTCTTCTTGGCTTCATGCAC-3’
IL6	5’-AAATGTCGAGGCTGTGCAGA-3’	5’-TCCACTCGTTCTGTGACTGC-3’
IL9	5’-CGAAGCCAGAACTGTCCCTG-5’	5’-GTTGCATGGCTGTTCACAGG-3’
TGFB1	5’-TGAACCCAAGGGCTACCATG-3’	5’-GTGCTGGTTGTACAGAGCCA-3’

Sus scrofa (pig) sequences for CCL2 (chemokine [C-C motif] ligand 2), COL3A1 (collagen 3A1), CXCL8 (chemokine [C-X-C motif] ligand 8, also called interleukin-8), GAPDH (glyceraldehyde-3-phosphate dehydrogenase), IL2 (interleukin 2), IL4, IL9, and TGFB1 (transforming growth factor beta-1).

### Statistics

Statistical analysis was performed using the non-parametric t-test and one-way ANOVA and the Sidak test with SPSS Statistics, Version 22 software and GraphPad Prism Version 6 (GraphPad Prism Software, Inc. San Diego, CA, USA). Significance was considered at p<0.05. Error bars represent standard error.

## Results and discussion

### Captopril treatment improves survival and blood cell recovery after total body irradiation

**Fig 1:**
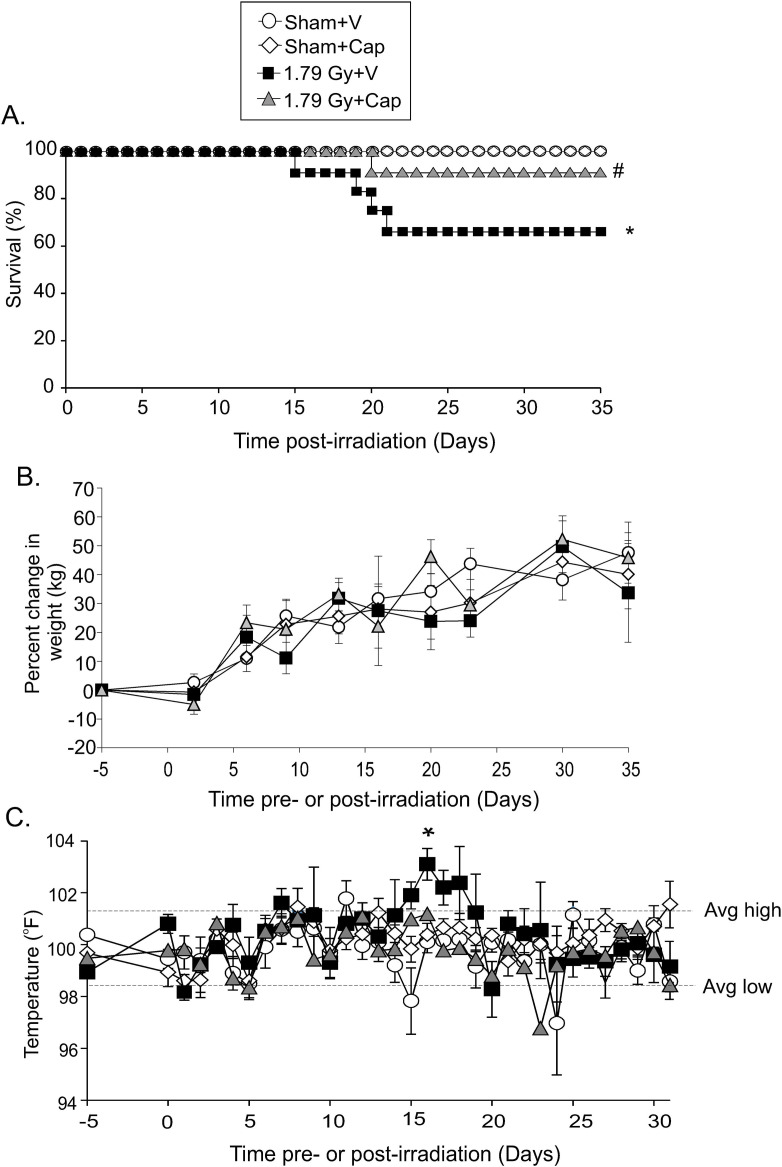
Effects of captopril treatment on survival, weight gain, and temperature in Göttingen minipigs. Göttingen minipigs, 4–6 months of age, were exposed to 1.79 Gy total body ^60^Co irradiation or sham irradiated. Minipigs either received vehicle (V, water) or captopril (Cap), provided orally twice daily starting 4 h post-irradiation through 12 days post-irradiation. A. Kaplan-Meier Curve of the effect of captopril on survival from total body irradiation. * indicates p<0.05 compared with all other groups; # indicates p<0.05 compared with vehicle-treated survival. B. Baseline weights for all animals were obtained at 5 days prior to irradiation (day -5). Weights were obtained on the indicated dates. Graphs show mean weight gain, ± SEM. C. Baseline temperatures were obtained at 5 days prior to irradiation (day -5). Temperatures were obtained from animals on the indicated dates. Graphs show mean weight gain, ± SEM. Weights and temperatures include all animals extant at the time point.

Previous studies indicated that the half lethal dose at 30 days (LD_50/30_) for Göttingen minipigs occurred at ~1.78–1.79 Gy total body irradiation [10]. No adverse effects were observed in sham-irradiated animals receiving either vehicle or captopril for 12 days. Captopril treatment was initiated 4 h following radiation exposure through 12 days post-irradiation. At 1.79 Gy (0.485–0.502 Gy/min), mortality was observed between 15–21 days post-irradiation (Fig 1A). We observed ~33% mortality at 21 days post-irradiation in minipigs treated with vehicle (Fig 1A). In contrast, only 9% mortality was observed in captopril-treated minipigs (Fig 1A, p<0.05 compared with vehicle-treated animals).

Our data showed that animals in all groups continued to show increase in body weight following total body irradiation, and significant decreases in body weight were not observed prior to early euthanasia (days 15–21) (Fig 1B). We observed a trend toward lower body weight in vehicle treated animals that survived to 35 days compared with captopril treated animals. Animals were also monitored for body temperature. Petechiae was not consistently observed, but if present could be observed between 5–12 days post-irradiation; petechiae was not a good indicator of early mortality vs survival at 35 days. We observed a significant increase in temperature among the vehicle-treated irradiated animals on day 16 post-irradiation (Fig 1C). This increase was not seen in irradiated animals that received captopril.

Diffuse hemorrhage has been described previously in Göttingen minipigs requiring early euthanasia following total body irradiation [7]. In our study, diffuse hemorrhage was observed in ~50% of animals requiring early euthanasia, one at 19 days and one at 20 days post-irradiation. Interestingly, no captopril-treated animals exhibited diffuse hemorrhage at the time of necropsy. Other animals that were euthanized at early endpoints exhibited lethargy, ataxia, or anorexia. Together, these findings are in agreement with previous findings from our laboratory and others using murine models to demonstrate that ACE inhibitors improves survival from H-ARS following total body irradiation [14–17, 21, 22].

**Fig 2:**
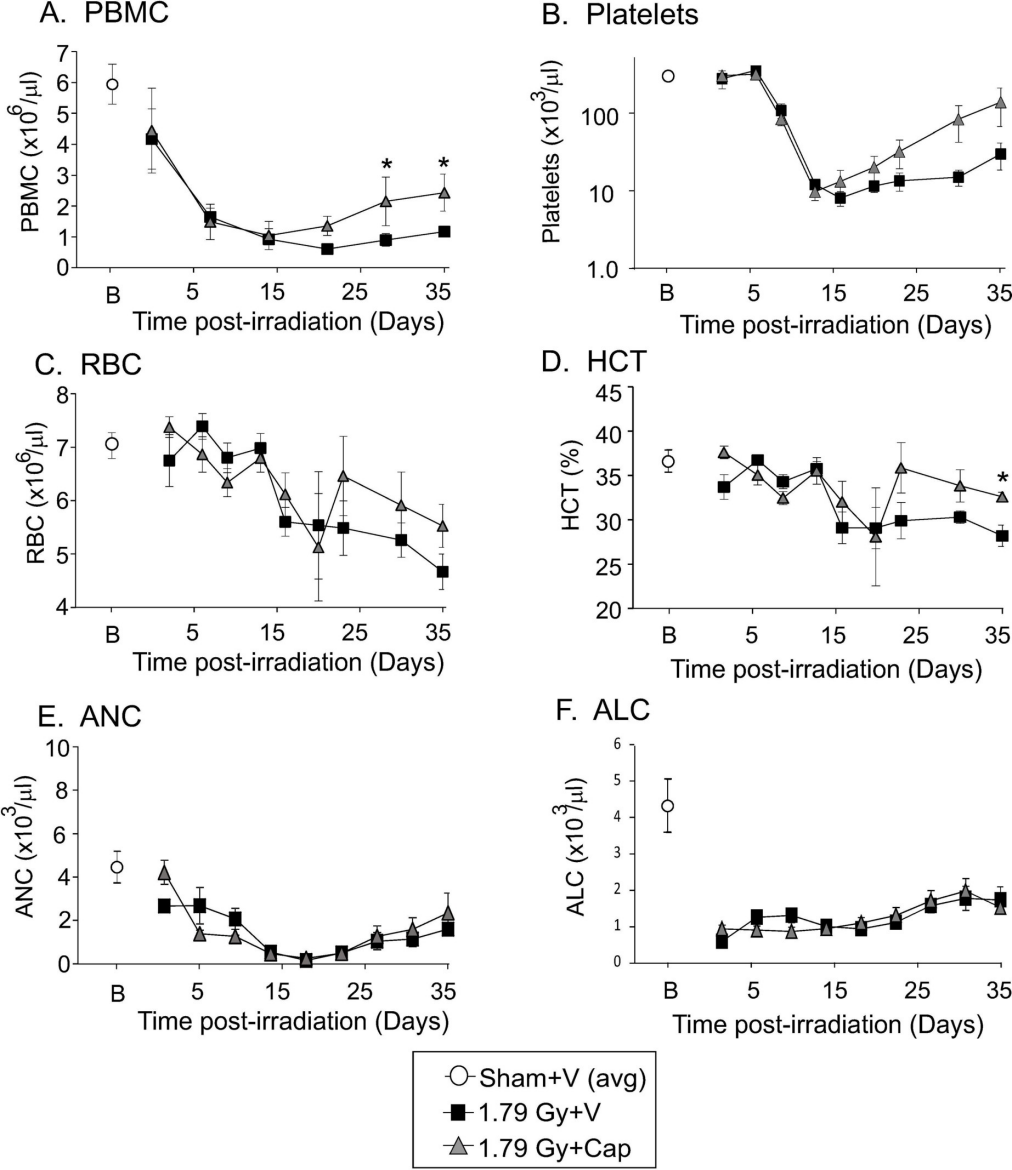
Effect of delayed captopril treatment on mature blood cell loss and recovery after total body irradiation. Göttingen minipigs, 4–6 months of age, were exposed to 1.79 Gy total body ^60^Co irradiation or sham irradiated. Minipigs either received vehicle (V, water) or captopril (Cap), provided orally twice daily starting 4 h post-irradiation through 12 days post-irradiation. Blood was obtained on day -3 (pre-irradiation), and on the indicated time points post-irradiation for analysis and quantification of **A.** red blood cells (RBC), **B.** platelets, **C.** red blood cells (RBC), **D.** hematocrit (HCT), **E.** absolute neutrophil count (ANC), and **F.** absolute lymphocyte count (ALC). Data show means ± standard error of the mean, n = 3–4 pigs per group. Average blood cell levels for sham irradiated animals are shown at B (basal) for clarity. * p < 0.05 for captopril treatment vs radiation + vehicle.

We investigated the effect of captopril on survival and blood cell recovery in the Göttingen minipig model of radiation injury. Blood cell analysis showed that captopril treatment improved the recovery of peripheral blood mononuclear cells (PBMC) as a combined population at 27 and 35 days post-irradiation (p<0.05; Fig 2A). We also observed evidence of improved recovery of platelets and red blood cells (RBCs) at 27 and 35 days post-irradiation (Fig 2B and 2C, respectively). Significant improvement was observed in hematocrit (HCT) at 35 days post-irradiation in captopril-treated minipigs vs vehicle treated animals (p<0.05; Fig 2D). This data was in agreement with findings from our laboratory and others showing that improved survival from total body irradiation in mice was associated with improved recovery of platelets, RBC, reticulocytes, and HCT [15, 16]. We did not observe significant differences in the levels of absolute neutrophils (ANC) or absolute lymphocyte counts (ALC) between vehicle-treated and captopril treated groups (Fig 2E and 2F, respectively).

**Fig 3:**
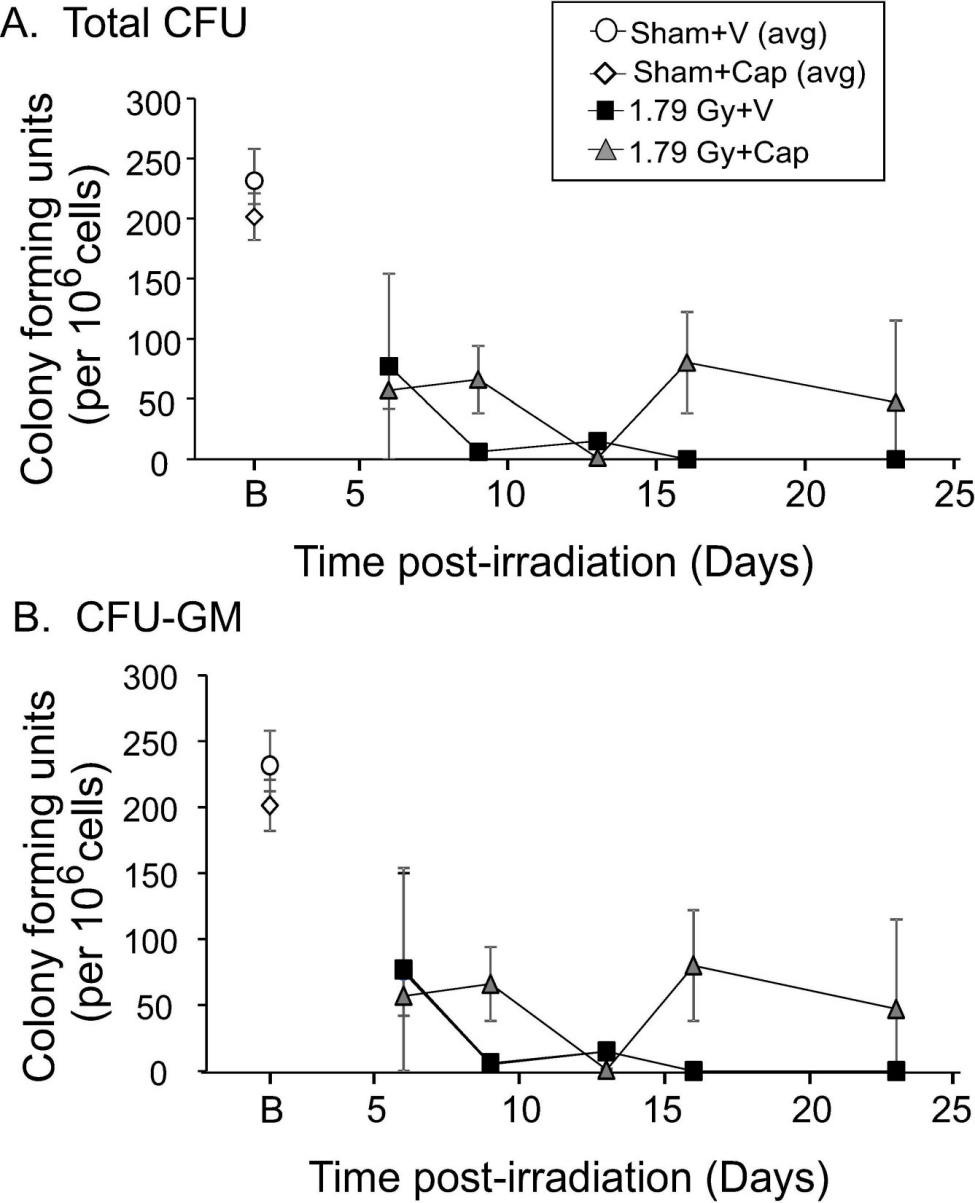
Effect of delayed captopril treatment on circulating hematopoietic progenitors after total body irradiation. Göttingen minipigs, 4–6 months of age, were exposed to 1.79 Gy total body ^60^Co irradiation or sham irradiated. Minipigs either received vehicle (V, water) or captopril (Cap), provided orally twice daily starting 4 h post-irradiation through 12 days post-irradiation. Blood was obtained on day -3 (pre-irradiation, shown at B), and on days 6, 9, 13, 16, and 23 post-irradiation for analysis and quantification of **A.** total colony forming units (CFU) and **B.** granulocyte-macrophage colony forming units (CFU-CM). Average blood cell levels for sham irradiated animals are shown at B (basal) for clarity. Data show means ± standard error of the mean, n = 3–4 pigs per group.

We investigated the effects of captopril on circulating hematopoietic progenitors which were previously shown to decline following total body irradiation in minipigs [7, 9, 10]. Captopril treatment alone did not significantly affect circulating hematopoietic progenitors (Fig 3). Total body irradiation resulted in a decline in progenitors from 7 through 23 days post-irradiation (Fig 3). We observed a trend toward improved total CFU and CFU-GM in captopril treated animals versus vehicle at 9 and 16 days post-irradiation, but this did not reach significance due to large differences within the captopril group. Over the time course examined, the circulating hematopoietic progenitors did not recover to baseline levels.

**Fig 4:**
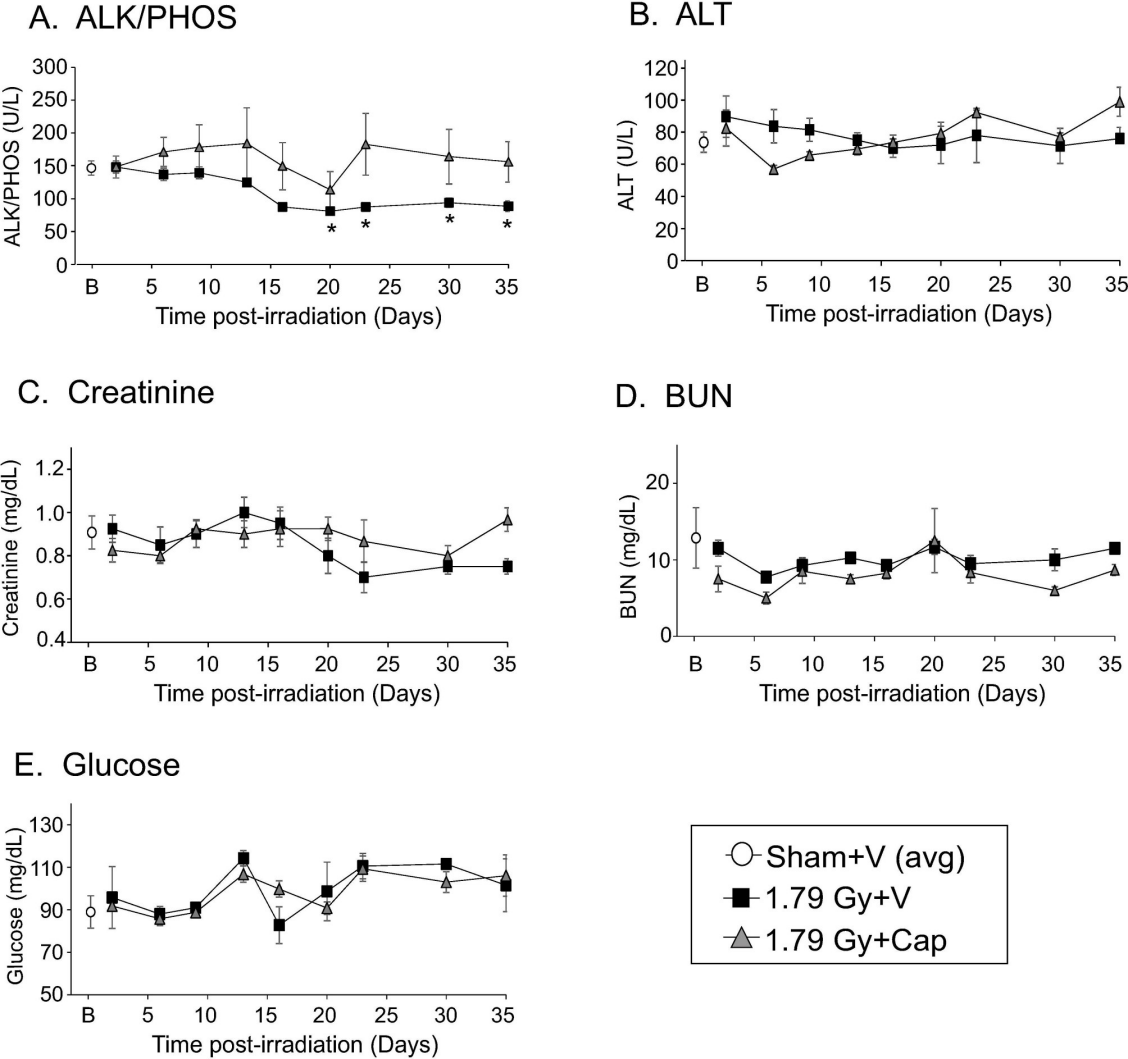
Effect of delayed captopril treatment on blood chemistry after total body irradiation. Göttingen minipigs, 4–6 months of age, were exposed to 1.79 Gy total body ^60^Co irradiation or sham-irradiation. Minipigs either received vehicle (V, water) or captopril (Cap), provided orally twice daily starting 4 h post-irradiation through 12 days post-irradiation. Blood was obtained on day -3 (pre-irradiation, shown at B), and on days 3, 6, 9, 13, 16, 20, 23, 30, and 35 post-irradiation for analysis and quantification of **A.** alanine aminotransferase (ALT), **B.** alkaline phosphatase (ALK/PHOS), **C.** creatinine, **D.** blood urea nitrogen (BUN), and **E.** glucose. Average blood cell levels for sham irradiated animals are shown at B (basal) for clarity. Data show means ± standard error of the mean, n = 3–4 pigs per group. * p < 0.05 difference from sham control.

Previous studies in Göttingen minipigs demonstrated that blood levels of alkaline phosphatase (ALK/PHOS) are reduced following total body irradiation [6]. Blood analyses were performed on animals in a time course following total body irradiation (Fig 4). Significant declines in ALK/PHOS from baseline were observed in vehicle-treated minipigs at 15–35 days post-irradiation (p<0.05; Fig 4A). Interestingly, no significant changes were observed in ALK/PHOS in captopril-treated irradiated animals compared with baseline (Fig 4A). We did not observe significant changes in alanine aminotransferase, creatinine, blood urea nitrogen, or glucose (Fig 4B–4E, respectively).

### Captopril mitigates radiation-induced inflammation

**Fig 5:**
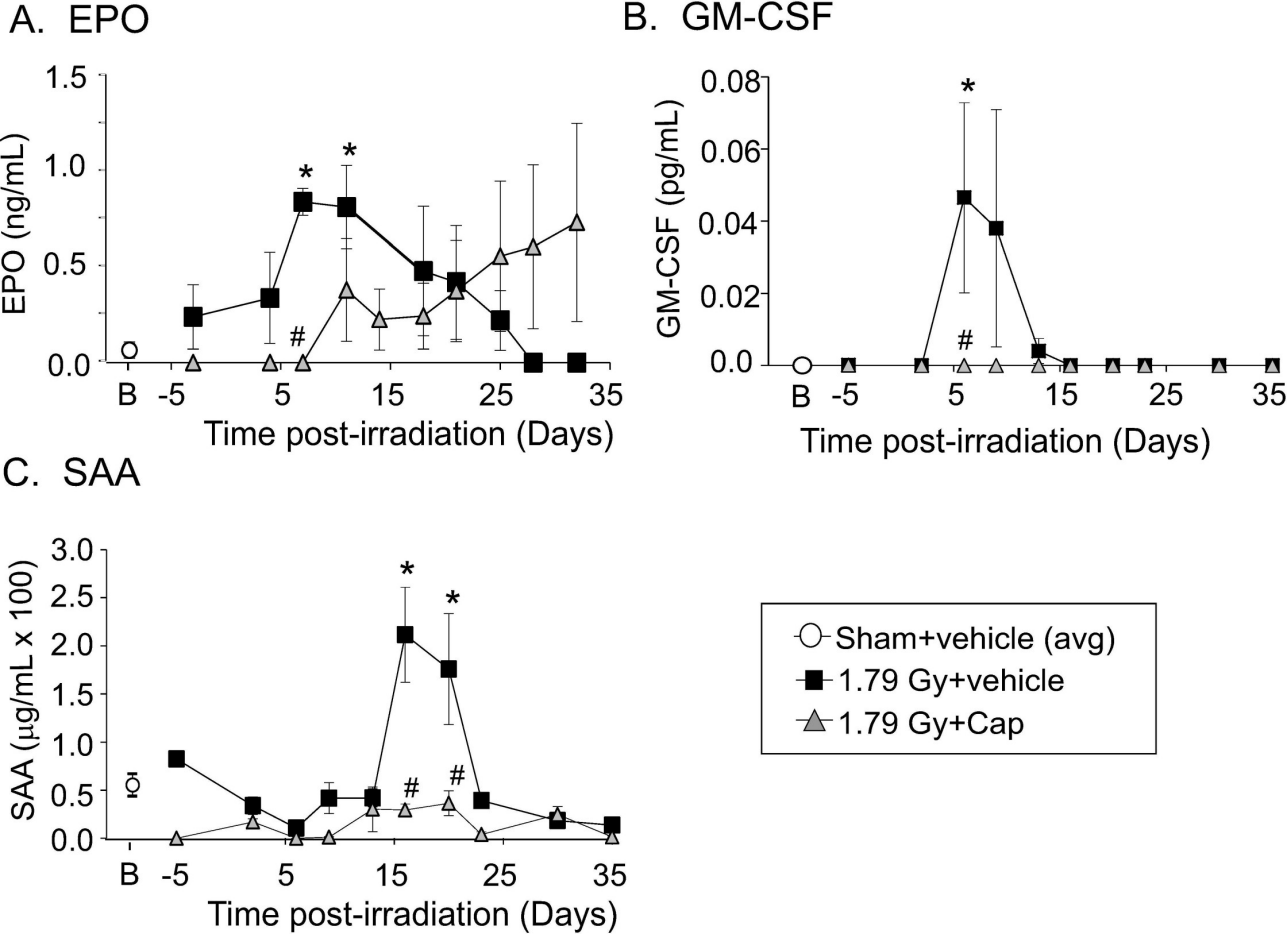
Effect of delayed captopril treatment on serum cytokine levels after total body irradiation. Göttingen minipigs, 4–6 months of age, were exposed to 1.79 Gy total body ^60^Co irradiation or sham-irradiation. Minipigs either received vehicle (V, water) or captopril (Cap), provided orally mixed with yogurt twice daily starting 4 h post-irradiation through 12 days post-irradiation. Serum was obtained on days pre- or post-irradiation for ELISA for **A.** erythropoietin (EPO), **B.** granulocyte-macrophage colony stimulating factor (GM-CSF), or **C.** serum amyloid A (SAA). Average blood cell levels for sham irradiated animals are shown at B (basal) for clarity. Data show means ± standard error of the mean, n = 4 pigs per group for all time points except the last two time points (27 and 35 days post-irradiation) which included only 3 animals for the 1.79 Gy, vehicle-treated group. * p < 0.05 difference from sham control. # p < 0.05 difference from radiation + vehicle.

We previously showed that captopril treatment post-irradiation mitigated radiation-induced acute inflammatory response and the upregulation of several hematopoietic cytokines in a murine model of H-ARS [14–16]. We investigated radiation-induced levels of the hematopoietic cytokines erythropoietin (EPO) and granulocyte-macrophage colony stimulating factor (GM-CSF), and the acute inflammatory response protein serum amyloid protein A (SAA) in the serum by ELISA in a time course following total body irradiation. EPO was significantly increased ~6-fold in vehicle treated animals at 7 and 11 days post-irradiation (Fig 5A). We observed a trend toward increased EPO through day 21 post-irradiation in the vehicle-treated group, followed by a reduction of EPO to below basal levels at days 28 and 32 post-irradiation. Captopril significantly suppressed the radiation-induced increase of EPO at 7 days, to below baseline levels. We observed a trend toward increased EPO in the captopril-treated animals, from 11 through 32 days post-irradiation. GM-CSF was also elevated in vehicle-treated irradiated animals on day 7 post-irradiation (Fig 5B). Captopril significantly reduced radiation-induced GM-CSF, and this cytokine remained at basal levels throughout the time course. SAA was elevated in vehicle-treated irradiated animals ~4-fold on days 16 and 20 post-irradiation before returning to baseline (Fig 5C). Captopril treatment significantly reduced SAA increases at both time points, and SAA remained near basal levels.

### Captopril treatment normalizes cytokine expression in the bone marrow

**Fig 6:**
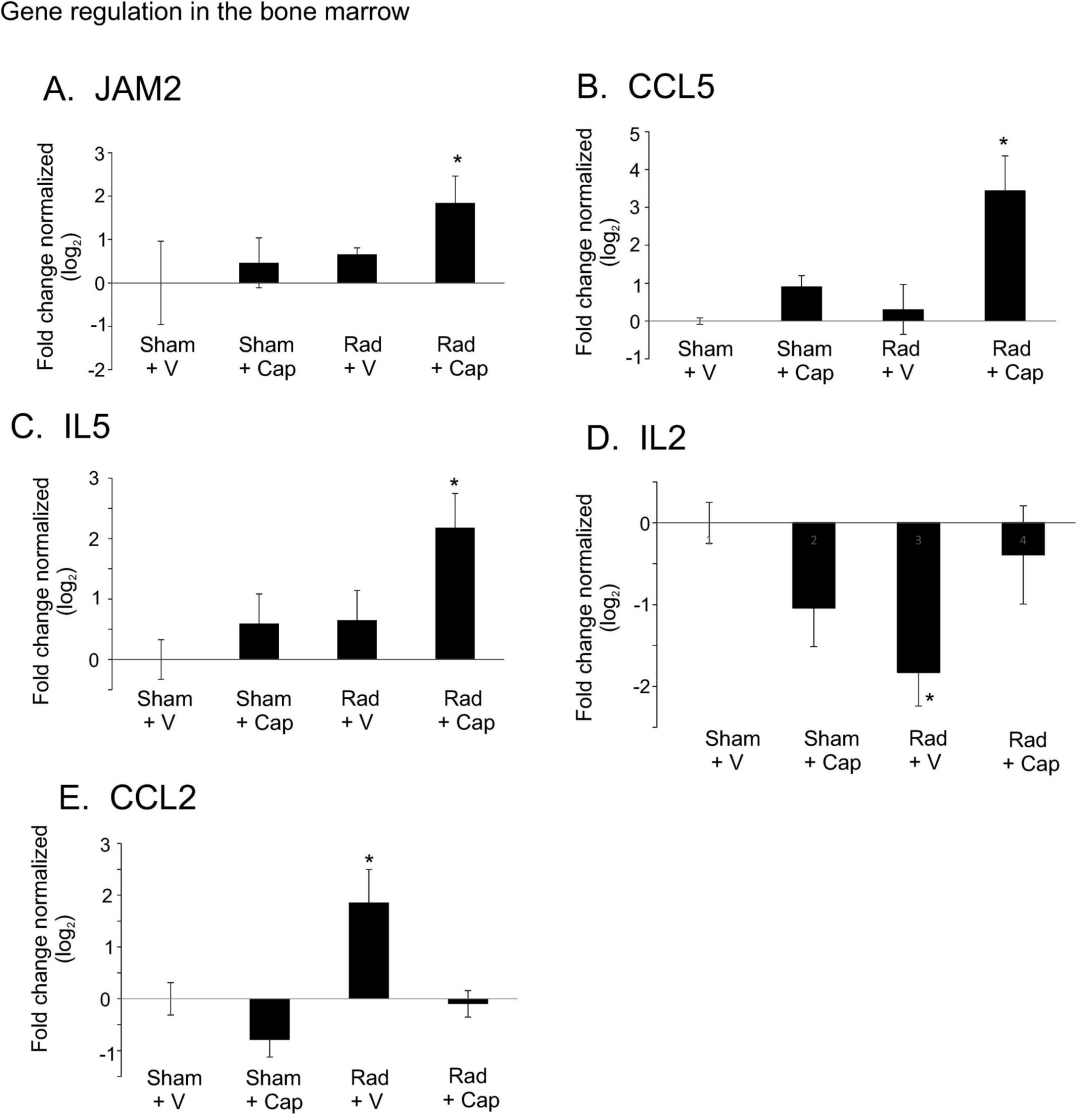
Effect of delayed captopril treatment on cytokine gene expression in the bone marrow after total body irradiation. Göttingen minipigs, 4–6 months of age, were exposed to 1.79 Gy total body ^60^Co irradiation or sham irradiation. Minipigs either received vehicle (V, water) or captopril (Cap), provided orally twice daily starting 4 h post-irradiation through 12 days post-irradiation. Bone marrow tissue was obtained at the time of euthanasia. All sham irradiated animals were euthanized at 32–35 days after sham irradiation. Radiation + V animals were euthanized at 17, 21, 34 and 35 days post-irradiation. Radiation + captopril animals were euthanized at 21 and 33, 34 and 35 days post-irradiation. RNA was prepared from bone marrow tissue and used for qRT-PCR for **A.** JAM2 (junctional adhesion molecule B), **B.** CCL5 (C-C motif chemokine ligand 5), **C.** IL5 (interleukin 5), **D.** IL2 (interleukin 2), and **E.** CCL2 (C-C motif chemokine ligand 2). Graph shows means of gene expression normalized to GAPDH ± standard error of the mean, n = 4 pigs per group. * p < 0.05 difference from sham control.

Our previous studies showed that captopril treatment improved bone marrow cellularity and recovery [14]. At the time of collection, the bone marrow of irradiated animals was scleriotic with loss of bone marrow elements suggesting reduced bone marrow cellularity and this caused difficulty in tissue collection as noted by our veterinary pathologist. We investigated gene expression of cytokines and factors as indicators of bone marrow stromal function. *JAM2*, a marker for bone marrow stromal cells that retain hematopoietic stem and progenitor cells in the bone marrow, was increased in captopril-treated irradiated animals compared with all other groups (p<0.05, Fig 6A). *CCL5*, a cytokine produced by bone marrow fibroblast reticular cell-like cells to support platelet production, was also increased only in captopril-treated irradiated minipigs (p<0.05, Fig 6B). Likewise, *IL5*, a cytokine secreted by CD34^+^ hematopoietic progenitors and CD4^+^ T cells that supports eosinophil differentiation, proliferation and maturation, was increased in captopril-irradiated minipigs compared with all other groups (p<0.05; Fig 6C). *IL2*, a cytokine produced by bone marrow mononuclear cells essential for lymphocyte proliferation and for bone marrow erythropoiesis, was significantly impaired in irradiation + vehicle animals (p<0.05), but returned to basal levels with captopril treatment (Fig 6D). Finally, *CCL2*, a cytokine produced by bone marrow stromal cells to recruit macrophages, was significantly increased in irradiation + vehicle animals (p<0.05), but was suppressed to basal levels in captopril treated animals (Fig 6E). We did not observe significant changes in *COL3A1*, *CXCL8*, *IL6*, *IL9*, *IL10*, or *TGFB1* expression in the bone marrow (data not shown). Overall, these data suggest a normalization of bone marrow cellular function by captopril, with increased support for platelet production, eosinophil proliferation, and erythropoiesis.

## Conclusions

The development of radiation countermeasures for a mass casualty event to mitigate acute radiation syndrome remains a medical challenge. The development of radiation countermeasures under the FDA Animal Rule requires demonstration of efficacy in two animal models, one of which must be a non-rodent [5]. In recent years, the minipig model of H-ARS has received increased attention [11]. This model recapitulates many of the characteristics of H-ARS in humans, and experiments demonstrate that previously identified radiation countermeasures are effective in minipigs [8, 23]. Our previous studies investigated the activity of the ACE inhibitor captopril as an H-ARS countermeasure in several murine model systems [14]. Here we demonstrate that the ACE inhibitor captopril improves mature blood cell recovery, reduces radiation-induced cytokine production and inflammation, and normalizes gene expression in the bone marrow when administered for 12 consecutive days after radiation exposure in minipigs.

We previously demonstrated that captopril improved survival from H-ARS in mice; survival correlated with improved bone marrow recovery and improved mature blood cell recovery [14, 15]. Our current findings show that in Göttingen minipigs, captopril also improved recovery of mature blood cells through 35 days post-irradiation, with significant improvement in peripheral mononuclear cells. We also observed a trend toward improved platelets and RBC. The improvement in RBC was reflected by a significant recovery of overall hematocrit in minipigs treated with captopril. We also observed a trend toward improved recovery of circulating hematopoietic progenitors. Our work and others have shown that in mice exposed to an ~LD_50/30_ dose of radiation, many mature blood cells recover significantly by 30 days post-irradiation [16, 24]. In contrast, recovery of mature blood cells in Göttingen minipigs required more than 56 days post-irradiation [25]. Future studies will investigate a longer time course of hematopoietic recovery by captopril.

Our previous studies in mice also showed that captopril countermeasure activity correlated with the suppression of radiation-induced cytokine expression, especially EPO, G-CSF, and SAA [14, 15]. A recent study by Chopra et al. of gene expression profiles in the liver, lung, and hearts of irradiated Göttingen minipigs showed that these three organs displayed non-overlapping differences in gene expression when comparing non-surviving animals with surviving animals or control animals. Interestingly, upregulation of EPO, SAA2 and SAA3 were in the top 10 most significantly induced mRNAs in the liver samples of non-surviving animals compared with either surviving animals or control animals [26]. One caveat of the study by Chopra et al. was that SAA and EPO upregulation in surviving animals was not measured at the same time points as in the non-surviving animals, but instead was measured later after acute inflammation had resolved. For all vehicle-treated animals, we observed increases in SAA and EPO, but only half of these animals required early euthanasia. This suggests that SAA and EPO are upregulated following total body irradiation in an inflammatory phase, but these cytokines may not be a good indicator of early moribundity vs survival. Our current findings also show that captopril significantly suppressed radiation-induced levels of EPO (at early time points), GM-CSF, and SAA in the Göttingen minipigs. Interestingly, the dates for increased SAA correlate with the times of increased temperatures in the radiation + vehicle group of animals. Together, our data on radiation-induced cytokine expression recapitulates our findings in the murine model, and provides further evidence of the importance of the radiation-induced cytokine expression in morbidity and mortality post-irradiation.

The expression of high levels of GM-CSF or other cytokines following total body irradiation are hypothesized to partly function to boost stem and progenitor cell proliferation in the bone marrow to replenish mature blood cell populations. However, some studies suggest that the acute expression of cytokines may be linked to hematopoietic stem cell pool exhaustion by forcing the proliferation of cells that may not have completed DNA repair [27]. Additionally, a recent report suggests that acute inflammatory response, especially SAA, following total body irradiation is correlated with poor survival [26]. Our current study shows that the time course of high levels of cytokine expression post-irradiation correlates with the onset of morbidity in the minipigs. Our findings show that while elevated levels of SAA correlate with the time point of morbidity, it is not an absolute predictor of mortality, since all vehicle-treated irradiated animals displayed elevated SAA, but not all of them required early euthanasia. Our findings using murine models of H-ARS indicate that captopril suppression of acute cytokine expression following total body radiation is correlated with improved recovery of the bone marrow stem and progenitor cells. We hypothesize that suppression of early radiation-induced cytokines allows recovery of bone marrow stem and/or progenitor cells from DNA damage prior to their re-entry into the cell cycle. The effect of captopril on stem and progenitor cells following total body irradiation is an area that we are currently investigating further.

In summary, our data indicates that captopril, when administered post-irradiation, acts as a mitigator against H-ARS in the Göttingen minipig model. Further work is needed to understand the mechanism(s) by which captopril exerts its effects on hematopoietic progenitors to protect them from radiation injury as well as the mechanism(s) by which captopril suppresses radiation-induced cytokine expression.

## Supporting information

S1 File Percent changes in weights and temps Gottingen minipigs. This is a data file.
(PDF)

S2 File qPCR data. This is a data file.
(PDF)

S3 File Hematopoietic progenitor count. This is a data file.
(PDF)

S4 File ELISA data. This is a data file.
(PDF)

S5 File Gottingen minipig CBC and blood chemistry. This is a data file.
(PDF)
