# Supplementary material for: Effects of captopril against radiation injuries in the Göttingen minipig model of hematopoietic-acute radiation syndrome
Source: PLoS One. 2021 Aug 27;16(8):e0256208. doi: 10.1371/journal.pone.0256208 (PMC8396780; doi:10.1371/journal.pone.0256208)
Supplement: S1 File — This is a data file. (PDF) [file pone.0256208.s001.pdf]

changes in weights

lbs	animal #	-14D	-5D	0D	2	6
SHAM + V	6402	19.1	23		24	28
	3403	20.9	25		26.9	25
	2164	23.1	25		24	29
SHAM + CAP	4043	17.6	21		23.8	20
	3284	17.6	19		17	17
	6697	19.4	23		23	22
	6461	23.8	24		26	28
RAD + V	6496	20.7	25		24	24
	5091	21.1	24		23.8	20
	6930	20.9	25		26	26
	6689	20.5	25		23.7	17
RAD + CAP	7472	22	25.5		22.5	25
	5902	19.4	24		25	26
	7090	20.7	23		21.5	24
	7227	21.1	25.5		24	25

lbs		-14D	-5D	0D	+2D	+6D
RAD + V	106 0406	23.8	23			35.2
	106 0520	22.3	23			33.6
	106 0121	22.9	21			30
	106 0066	22.9	25			32.2
RAD + CAP	106 0783	20.9	20			29.8
	302 8852	22.1	22			33
	302 8127	24.7	23			29.4
	302 8941	22.9	20			32.8

kg		-14D	-5D	0D	+2D	+6D
RAD + V	1104 896	8.3	8.5			10.2
	1103 407	7.9	8.4			9.7
	1104 381	7.4	8.3			9.7
	1103 849	8.7	8.6			10.6
RAD + CAP	1103 491	7.7	7.9			9
	1104 462	8.3	8.6			10.2
	1103 181	8.2	8.4			10.1

9	13	16	20	23	30	35
26	27.5	31	30	32	33	31
32	35	35	37	39	39	43
34	30	30	31	34	30	34
25	25	26	26	29	28	27
20.5	24	23	24	25	26	25
25	26	27	25	26	27	28
30.5	33	35	35	35	37	40
28.5	29.5	23	24			
27	27	24				
30.5	26	23	25	33	33	26
24	26	28	28	29	29	24
31	31	33	31			
20	32	32	32	33	31	30
18	20	23	28	27	26	25
21.5	31	32	33	34	31	35

+9D	+13D	+16D	+20D	+23D	+30D	+35D
	37.6		42.2		42.8	39
	33.8					
	31.4		35.4		36.3	35
	32.8		36		37.4	33
	30.2		33		36	34
	33.6		37		39.8	36
	29.4		34.4		37.6	33
	33		37		39.4	35

+9D	+13D	+16D	+20D	+23D	+30D	+35D
	11.1		11.5		11.7	11.9
	10.9		11.5		11.8	12.1
	10.8					
	11.7		12.1		12.1	12.4
	9.9		10.5		10.6	11.2
	11.4		12.3		12.2	12.6
	11.3		12		12.2	12.8

changes in temperature

	animal #	-5D	0D	+1D	+2D	+3D	+4D	+5D	+6D	+7D	+8D	+9D	+10D	+11D
SHAM + V	6402	100.7	99.2	101.9	99.6	101.1	100.3	98.9	101.1	101.3	101.3	100.8	100.3	101.9
	3403	100.2	100.7	99.0	97.8	100.3	97.2	96.5	97.2	98.8	98.5	100.4	96.6	99.8
	2164	99.9	99.1	98.9	100.0	99.5	100.0	98.7	100.2	101.3	101.1	100.6	99.7	103.7
	3951	100.7	98.8	99.0	100.2	99.9	98.1	99.9	101.1	100.9	101.1	100.7	102.1	101.7
SHAM + CAP	4043	99.4	97.1	97.8	97.2	100.9	100.0	99.8	101.1	102.4	102.9	101.8	100.6	100.6
	3284	97.9	99.9	98.6	99.8	99.8	100.0	98.3	99.9	99.7	100.5	100.1	100.1	100.7
	6697	100.1	99.6	98.9	100.1	101.8	101.4	99.7	102.1	100.9	102.8	102.1	100.2	100.8
	6461	101.3	99.1	99.1	97.4	99.8	98.5	96.6	99.2	99.4	99.6	99.5	98.2	98.8
RAD + V	6496	99.1	101.9	99.0	99.1	99.2	101.4	100.2	101.2	102.0	101.1	101.9	100.7	100.5
	5091	98.4	100.8	97.3	98.5	99.0	101.7	98.6	99.9	101.1	100.5	100.8	98.6	101.1
	6930	100.0	101.7	100.6	100.4	101.7	100.7	100.3	102.1	102.2	102.1	102.5	101.2	102.1
	6689	98.2	98.8	95.8	98.9	99.7	99.2	98.1	98.9	101.1	100.0	99.3	96.7	99.5
RAD + CAP	7472	99.4	100.5	100.1	101.3	100.9	100.8	99.7	101.3	102.1	101.5	100.5	101.1	101.7
	5902	98.9	100.5	98.9	95.8	100.3	97.0	96.0	98.9	99.1	100.2	93.3	98.0	98.2
	7090	99.6	99.0	99.7	100.7	101.0	99.7	96.9	100.6	101.3	101.8	103.4	100.1	100.7
	7227	100.0	99.1	100.6	99.2	101.1	97.3	100.8	101.2	100.3	100.6	100.5	99.3	101.4
RAD + V	106 0406	101								101.4				
	106 0520	100.1								101.2				
	106 0121	101.1								100.8				
	106 0066	100.1								101.1				
RAD + CAP	106 0783	101.1								101.6				
	302 8852	99.3								100.8				
	302 8127	99								100.5				
	302 8941	101.5								101.6				
RAD + V	1104 896	100.4								101.8				
	1103 407	100.2								101.6				
	1104 381	99.1								99.8				
	1103 849	95.8								99.8				
RAD + CAP	1103 491	101.2								99.7				
	1104 462	98.7								99.6				
	1103 181	98.5								101.1				

+12D	+13D	+14D	+15D	+16D	+17D	+18D	+19D	+20D	+21D	+22D	+23D	+24D	+25D	+26D	+27D
100.6	100.5	100.9	100.2	100.7	101.3	101.7	98.9	100.8	101.1	100.1	100.4	99.7	102.0	100.8	100.1
99.2	99.0	97.5	93.6	98.8	98.3	100.0	101.7	98.9	100.3	98.7	98.2	90.1	99.4	98.8	97.4
101.3	99.7	99.9	98.1	100.0	100.0	100.2	98.9	100.5	99.9	99.6	100.1	98.8	101.6	100.9	99.2
98.7	101.2	98.5	99.4	100.9	101.0	98.9	97.1	100.1	99.4	99.0	101.3	99.3	101.6	100.1	97.4
100.3	101.8	100.9	100.4	100.5	100.7	101.7	100.7	99.7	99.3	100.8	100.1	100.4	101.3	101.4	101.7
100.6	101.7	100.5	99.9	100.8	101.2	100.5	100.3	100.9	99.9	100.1	100.1	99.5	99.6	101.0	101.0
101.6	102.1	101.0	100.8	101.3	101.2	101.5	101.1	100.9	100.6	101.3	102.0	101.2	100.9	100.8	101.6
99.1	99.3	99.3	98.2	98.9	99.5	98.9	98.8	99.9	97.6	98.6	97.9	97.6	98.4	98.2	99.5
101.1	100.8	103.4	103.7	103.3	103.6	103.1	102.5	104.1	103.0						
100.2	99.5	101.3	103.6	104.0	101.1	104.3	102.6	89.1							
102.6	101.6	100.8	101.0	103.5	103.8	102.5	100.9	99.9	100.9	101.2	101.3	99.6	99.4	99.8	100.1
100.1	99.3	99.0	99.3	101.6	100.3	99.6	98.9	100.0	98.5	99.6	99.8	98.9	99.5	99.2	98.6
102.8	101.2	104.0	102.7	102.9	101.8	104.1	102.7	100.3							
99.6	96.6	96.6	100.1	99.8	98.1	96.3	94.6	95.1	99.8	96.4	91.5	95.1	97.9	98.7	98.0
101.6	100.0	100.4	100.9	100.3	99.9	100.2	100.7	100.1	98.6	100.0	99.5	101.6	101.3	100.4	100.0
100.3	101.3	98.3	100.2	101.7	99.3	98.9	99.8	99.7	101.1	101.0	99.3	100.9	99.9	100.4	100.7
		102.6							100.2						
		102.9													
		101.8							101.3						
		102							101.1						
		101.3							101.1						
		101.1							100.6						
		101.1							101.6						
		101.8							100.9						
		101.1							101.4						
		101.6							100.2						
		99.3													
		100							100.7						
		100.6							100.5						
		101.3							101						
		101.6							101.9						

+28D	+29D	+30D	+31D	+35D
100.3	100.3	100.9	99.9	99.5
100.9	97.4	100.1	96.3	101.1
100.4	99.4	100.6	99.3	100.6
98.0	98.9	101.5	98.8	101.2
101.2	100.1	100.7	101.6	98.0
100.3	100.0	101.0	101.0	101.0
100.9	100.8	102.8	104.3	98.0
99.7	98.5	98.3	99.3	98.2
100.0	99.2	100.5	99.0	100.6
99.6	100.9	98.7	99.3	100.6
99.6	99.4	96.9	95.7	99.3
100.7	101.4	100.2	99.5	99.4
101.3	101.2	102.0	100.1	101.8
		100.9		
		101.8		
		101.9		
		100.7		
		101.7		
		101.7		
		100.7		
		100.6		
		100.2		
		99.7		
		99		
		101.1		
		101.3		
