# Supplementary material for: Effects of captopril against radiation injuries in the Göttingen minipig model of hematopoietic-acute radiation syndrome
Source: PLoS One. 2021 Aug 27;16(8):e0256208. doi: 10.1371/journal.pone.0256208 (PMC8396780; doi:10.1371/journal.pone.0256208)
Supplement: S2 File — This is a data file. (PDF) [file pone.0256208.s002.pdf]

qPCR raw data JAM2, CCL5, IL5, IL2, CCL2

Well	Fluor	Target	cDNA sample	Cq	GAPDH Values	Normalize to GAPDH	Avg of controls
A01	SYBR	CCL2	1 = sham veh	29.12	23.303	5.814	5.176
A02	SYBR	CCL2	2= sham vehi	29.81	24.041	5.766	5.176
	SYBR	CCL2	7 = 6402 shar	28.07	23.927	4.142	5.176
A01	SYBR	CCL2	1	28.884	23.083	5.801	5.176
B01	SYBR	CCL2	2	29.554	24.341	5.213	5.176
E06	SYBR	CCL2	D1	28.262	23.942	4.320	5.176
A03	SYBR	CCL2	4=sham captc	30.57	24.053	6.521	5.176
A04	SYBR	CCL2	5=sham captc	30.11	23.635	6.479	5.176
A05	SYBR	CCL2	6=sham captc	31.23	23.716	7.518	5.176
A06	SYBR	CCL2	7=sham captc	28.91	24.158	4.757	5.176
C01	SYBR	CCL2	4	30.636	24.138	6.498	5.176
D01	SYBR	CCL2	5	29.983	23.518	6.465	5.176
E01	SYBR	CCL2	6	30.677	23.884	6.793	5.176
F01	SYBR	CCL2	7	28.967	24.055	4.912	5.176
A07	SYBR	CCL2	8=radiation ve	26.33	22.767	3.567	5.176
A08	SYBR	CCL2	9=radiation ve	29.09	23.825	5.269	5.176
A09	SYBR	CCL2	10=radiation v	28.36	24.217	4.148	5.176
	SYBR	CCL2	8 = 5091 radia	26.95	26.136	0.809	5.176
G01	SYBR	CCL2	8	26.252	23.128	3.125	5.176
H01	SYBR	CCL2	9	29.260	23.818	5.442	5.176
A06	SYBR	CCL2	10	28.387	24.328	4.059	5.176
F06	SYBR	CCL2	D2	26.645	25.959	0.686	5.176
A10	SYBR	CCL2	11=radiaiton c	30.28	23.603	6.682	5.176
A11	SYBR	CCL2	12=radiation c	28.67	23.859	4.808	5.176
A12	SYBR	CCL2	13= radiation	29.64	24.132	5.507	5.176
	SYBR	CCL2	9 = 7227 radia	28.33	23.285	5.049	5.176
B06	SYBR	CCL2	11	29.545	23.567	5.979	5.176
C06	SYBR	CCL2	12	28.550	23.935	4.615	5.176
D06	SYBR	CCL2	13	29.455	23.990	5.465	5.176
G06	SYBR	CCL2	D3	27.691	23.056	4.635	5.176

E01	SYBR	IL2	1 = sham veh	33.92	23.303	10.618	9.980
E02	SYBR	IL2	2= sham vehi	33.21	24.041	9.168	9.980
	SYBR	IL2	7 = 6402 shar	34.04	23.927	10.111	9.980
A04	SYBR	IL2	1	33.648	23.083	10.565	9.980
B04	SYBR	IL2	2	34.447	24.341	10.106	9.980
E09	SYBR	IL2	D1	33.256	23.942	9.314	9.980

E03	SYBR	IL2	4=sham captc	33.94	24.053	9.890	9.980
E04	SYBR	IL2	5=sham captc	36.24	23.635	12.601	9.980
E05	SYBR	IL2	6=sham captc	36.75	23.716	13.031	9.980
E06	SYBR	IL2	7=sham captc	35.49	24.158	11.337	9.980
C04	SYBR	IL2	4				
D04	SYBR	IL2	5	33.583	23.518	10.065	9.980
E04	SYBR	IL2	6	36.230	23.884	12.346	9.980
F04	SYBR	IL2	7	34.814	24.055	10.759	9.980
E07	SYBR	IL2	8=radiation ve	35.01	22.767	12.246	9.980
E08	SYBR	IL2	9=radiation ve	35.80	23.825	11.978	9.980
E09	SYBR	IL2	10=radiation vehicle #6930				
	SYBR	IL2	8 = 5091 radiaition				
G04	SYBR	IL2	8	36.464	23.128	13.336	9.980
H04	SYBR	IL2	9	34.993	23.818	11.175	9.980
A09	SYBR	IL2	10	36.710	24.328	12.382	9.980
F09	SYBR	IL2	D2	36.385	25.959	10.427	9.980
E10	SYBR	IL2	11=radiaiton c	33.46	23.603	9.857	9.980
E11	SYBR	IL2	12=radiation c	33.49	23.859	9.632	9.980
E12	SYBR	IL2	13= radiation	34.74	24.132	10.613	9.980
	SYBR	IL2	9 = 7227 radia	35.32	23.285	12.039	9.980
B09	SYBR	IL2	11				
C09	SYBR	IL2	12	33.098	23.935	9.163	9.980
D09	SYBR	IL2	13	35.316	23.990	11.326	9.980

B01	SYBR	JAM	1 = sham veh	36.33	23.260	13.070	9.366
B02	SYBR	JAM	2= sham vehi	32.91	24.020	8.890	9.366
	SYBR	JAM	7 = 6402 shar	31.64	24.063	7.573	9.366
	SYBR	JAM	1 = sham veh	32.06	23.072	8.991	9.366
	SYBR	JAM	2= sham vehi	33.36	25.052	8.304	9.366
B03	SYBR	JAM	4=sham captc	36.08	23.952	12.128	9.366
B04	SYBR	JAM	5=sham captc	34.04	23.912	10.129	9.366
B05	SYBR	JAM	6=sham captc	34.50	24.117	10.379	9.366
B06	SYBR	JAM	7=sham captc	33.65	24.213	9.434	9.366
B07**	SYBR	JAM	8=radiation ve	31.45	22.996	8.454	9.366
B08	SYBR	JAM	9=radiation ve	33.10	24.201	8.900	9.366
B09	SYBR	JAM	10=radiation v	33.71	24.553	9.154	9.366
	SYBR	JAM	8 = 5091 radia	33.41	24.368	9.046	9.366
B10	SYBR	JAM	11=radiaiton c	31.66	23.942	7.719	9.366
B11	SYBR	JAM	12=radiation c	33.05	24.311	8.743	9.366
B12	SYBR	JAM	13= radiation	34.11	24.335	9.771	9.366
	SYBR	JAM	9 = 7227 radia	32.36	25.454	6.910	9.366

D01	SYBR	IL5	1 = sham veh	34.50	23.264	11.238	10.937
D02	SYBR	IL5	2= sham vehi	35.24	24.018	11.220	10.937
	SYBR	IL5	7 = 6402 shar	33.70	24.063	9.634	10.937

	SYBR	IL5	1 = sham veh	34.26	23.070	11.190	10.937
	SYBR	IL5	2= sham vehi	36.45	25.050	11.400	10.937
D03	SYBR	IL5	4=sham captc	36.06	23.952	12.104	10.937
D04	SYBR	IL5	5=sham captc	33.96	23.912	10.052	10.937
D05	SYBR	IL5	6=sham captc	34.32	24.117	10.207	10.937
D06	SYBR	IL5	7=sham captc	34.40	24.213	10.185	10.937
D07	SYBR	IL5	8=radiation ve	34.58	22.996	11.584	10.937
D08	SYBR	IL5	9=radiation ve	33.67	24.201	9.471	10.937
D09	SYBR	IL5	10=radiation v	34.09	24.553	9.539	10.937
	SYBR	IL5	8 = 5091 radia	34.35	24.368	9.979	10.937
D10	SYBR	IL5	11=radiaiton c	32.08	23.942	8.136	10.937
D11	SYBR	IL5	12=radiation c	33.35	24.311	9.042	10.937
D12	SYBR	IL5	13= radiation	34.35	24.335	10.013	10.937
	SYBR	IL5	9 = 7227 radia	32.86	25.454	7.402	10.937

G01	SYBR	CCL5	1 = sham veh	25.77	23.264	2.508	2.510
G02	SYBR	CCL5	2= sham vehi	26.44	24.018	2.424	2.510
	SYBR	CCL5	7 = 6402 shar	26.83	24.063	2.769	2.510
	SYBR	CCL5	1 = sham veh	25.32	23.070	2.250	2.510
	SYBR	CCL5	2= sham vehi	27.65	25.050	2.600	2.510
G03	SYBR	CCL5	4=sham captc	26.30	23.952	2.345	2.510
G04	SYBR	CCL5	5=sham captc	25.19	23.912	1.275	2.510
G05	SYBR	CCL5	6=sham captc	25.15	24.117	1.035	2.510
G06	SYBR	CCL5	7=sham captc	25.78	24.213	1.569	2.510
G07	SYBR	CCL5	8=radiation ve	25.08	22.996	2.086	2.510
G08	SYBR	CCL5	9=radiation ve	25.51	24.201	1.312	2.510
G09	SYBR	CCL5	10=radiation v	25.70	24.553	1.151	2.510
	SYBR	CCL5	8 = 5091 radia	28.40	24.368	4.028	2.510
H07	SYBR	CCL5	11=radiaiton c	22.95	23.942	-0.994	2.510
H08	SYBR	CCL5	12=radiation c	24.48	24.311	0.173	2.510
H09	SYBR	CCL5	13= radiation	25.01	24.335	0.673	2.510
	SYBR	CCL5	9 = 7227 radia	22.02	25.454	-3.438	2.510

Normalizedminus control		Fold change in expresssion	Fold change in expression w/ negatives	LOG BASE 2 INDIVIDUAL EXPRESSION		AVERAGE MINUS CONTROL	FOLD CHANGE AVERAGE EXPRESSION	LOG BASE 2 AVG EXPRESSION RESULTS FOR GRAPH
0.638		0.643	-1.556	-0.638		0.000	1.000	0.000
0.590		0.664	1.017	-0.590				
-1.034		2.047	1.000	1.034				
0.625		0.648	-1.542	-0.625				
0.037		0.975	1.017	-0.037				
-0.856		1.810	-0.552	0.856				
1.345	GRAPH	0.394	-2.541	-1.345		1.067	0.477317	-1.06698
1.303		0.405	-2.468	-1.303				
2.342		0.197	-5.069	-2.342				
-0.419		1.337	2.047	0.419				
1.322		0.400	-2.501	-1.322				
1.289		0.409	-2.444	-1.289				
1.618		0.326	-3.069	-1.618				
-0.264		1.201	1.148	0.264				
-1.609		3.050	4.668	1.609		-1.788	3.452862	1.787793
0.094		0.937	1.434	-0.094				
-1.028		2.040	3.122	1.028				
-4.367		20.628	10.060	4.367				
-2.051		4.145	3.963	2.051				
0.266		0.832	-1.202	-0.266				
-1.117		2.169	2.074	1.117				
-4.490		22.466	21.479	4.490				
1.506		0.352	-2.841	-1.506		0.167	0.890983	-0.166531
-0.368		1.291	1.975	0.368				
0.331		0.795	1.217	-0.331				
-0.127		1.092	-0.916	0.127				
0.803		0.573	-1.745	-0.803				
-0.561		1.475	1.410	0.561				
0.289		0.818	-1.222	-0.289				
-0.541		1.455	1.391	0.541				
0.637		0.643	-1.555	-0.637		0.000	1.000	0.000
-0.813		1.757	1.653	0.813				
0.131		0.913	1.000	-0.131				
0.585		0.667	-1.500	-0.585				
0.126		0.917	1.017	-0.126				
-0.666		1.587	-0.630	0.666				

-0.091		1.065	1.000	0.091	1.452	0.365398	-1.452459
2.621		0.163	-6.152	-2.621			
3.051		0.121	-8.286	-3.051			
1.356		0.391	-2.560	-1.356			
0.085		0.943	-1.061	-0.085			
2.366		0.194	-5.155	-2.366			
0.779		0.583	-1.716	-0.779			
2.266		0.208	-4.810	-2.266	1.944	0.259956	-1.943659
1.998		0.250	-3.994	-1.998			
3.356		0.098	-10.236	-3.356			
1.194		0.437	-2.288	-1.194			
2.402		0.189	-5.285	-2.402			
0.446		0.734	-1.362	-0.446			
-0.123		1.089	1.023	0.123	0.458	0.72792	-0.458148
-0.348		1.273	1.196	0.348			
0.633		0.645	-1.550	-0.633			
2.059	GRAPH	0.240	-4.167	-2.059			
-0.817		1.762	1.780	0.817			
1.346		0.393	-2.542	-1.346			
3.704		0.077	-13.035	-3.704	0.000	1.000	0.000
-0.476		1.391	1.269	0.476			
-1.793		3.464	1.000	1.793			
-0.375		1.296	-0.771	0.375			
-1.061		2.087	1.269	1.061			
2.762		0.147	-6.783	-2.762	1.152	0.450	-1.152
0.764		0.589	1.803	-0.764			
1.013		0.495	1.517	-1.013			
0.068		0.954	2.920	-0.068			
-0.912		1.881	1.145	0.912	-0.477	1.392	0.477
-0.466		1.381	4.228	0.466			
-0.212		1.158	3.546	0.212			
-0.320		1.248	-0.801	0.320			
-1.646		3.130	9.584	1.646	-1.080	2.114	1.080
-0.623		1.540	4.715	0.623			
0.405		0.755	2.313	-0.405			
-2.456	GRAPH	5.487	1.580	2.456			
0.301		0.811	-1.232	-0.301	0.000	1.000	0.000
0.284		0.821	1.007	-0.284			
-1.302		2.466	1.000	1.302			

0.253		0.839	-1.192	-0.253			
0.463		0.725	1.007	-0.463			
1.168		0.445	-2.247	-1.168	-0.299	1.231	0.299
-0.885		1.846	2.263	0.885			
-0.729		1.658	2.032	0.729			
-0.752		1.684	2.064	0.752			
0.647		0.639	-1.566	-0.647	-0.793	1.733	0.793
-1.465		2.761	3.384	1.465			
-1.398		2.635	3.229	1.398			
-0.958		1.942	-0.515	0.958			
-2.801		6.969	8.541	2.801	-2.288	4.885	2.288
-1.894		3.718	4.556	1.894			
-0.924		1.897	2.325	0.924			
-3.534	GRAPH	11.587	4.685	3.534			
-0.002		1.001	-0.999	0.002	0.000	1.000	0.000
-0.087		1.062	1.030	0.087			
0.259		0.836	1.000	-0.259			
-0.260		1.198	-0.835	0.260			
0.090		0.940	1.030	-0.090			
-0.165	GRAPH	1.121	1.090	0.165	-0.954	1.938	0.954
-1.235		2.354	2.290	1.235			
-1.476		2.781	2.705	1.476			
-0.941		1.920	1.868	0.941			
-0.424		1.342	1.003	0.424	-0.366	1.289	0.366
-1.198		2.295	2.232	1.198			
-1.359		2.565	2.495	1.359			
1.517		0.349	-2.863	-1.517			
-3.504		11.348	11.036	3.504	-3.407	10.606	3.407
-2.337		5.053	4.914	2.337			
-1.837		3.573	3.475	1.837			
-5.949		61.760	73.946	5.949			
