# Supplementary material for: Effects of captopril against radiation injuries in the Göttingen minipig model of hematopoietic-acute radiation syndrome
Source: PLoS One. 2021 Aug 27;16(8):e0256208. doi: 10.1371/journal.pone.0256208 (PMC8396780; doi:10.1371/journal.pone.0256208)
Supplement: S3 File — This is a data file. (PDF) [file pone.0256208.s003.pdf]

Hematopoietic progenitors

HPC counts per million plated

6 days post-irradiaiton

	CFU	GM	BFU
Sham			
6402	85	73	12
3403	105	101	4
2164		contaminated	
3951	101	92	9
Sham+Cap			
4043		contaminated	
3284	104	93	11
6697	10	10	0
6461	87	86	1
Rad			
6496	54	59	1
5091		contaminated	
6930	77	75	2
6689		contaminated	
Rad_Cap			
7472		contaminated	
5902	54	54	0
7090	82	78	4
7227	14	13	1

9 days post-irradiaiton

Sham	CFU	GM	BFU
6402	142	135	7
3403		contaminated	
2164	137	131	6
3951	114	111	3
Sham+Cap			
4043	128	127	1
3284	198	192	6
6697	195	191	4
6461	156	154	2
Rad			
6496		contaminated	
5091	10	9	1
6930	2	1	0
6689	2	1	1
Rad_Cap			
7472	11	11	0
5902	109	109	0
7090	121	119	2
7227	24	24	0

13 days post-irradiaiton

Sham	CFU	GM	BFU
6402	170	170	0
3403	257	255	2
2164	142	139	3
3951	213	211	2
Sham+Cap			
4043	227	224	3
3284	contaminated		
6697	132	127	5
6461	contaminated		

16 days post-irradiaiton

Sham	CFU	GM	BFU
6402	141	138	3
3403	140	136	4
2164	152	149	3
3951	120	118	2
Sham+Cap			
4043	170	163	7
3284	117	116	1
6697	contaminated		
6461	184	180	4

Rad			
6496	21	21	0
5091	0	0	0
6930	35	35	0
6689	5	5	0
Rad_Cap			
7472	0	0	0
5902	1	1	0
7090	2	2	0
7227	0	0	0

Rad			
6496	0	0	0
5091	0	0	0
6930	0	0	0
6689	0	0	0
Rad_Cap			
7472	50	50	0
5902	0	0	0
7090	110	108	2
7227	0	0	0

20 days post-irradiaiton

Sham	CFU	GM	BFU
6402	141	138	3
3403	140	136	4
2164	152	149	3
3951	120	118	2
Sham+Cap			
4043	170	163	7
3284	117	116	1
6697	contaminated		
6461	184	180	4
Rad			
6496	0	0	0
5091	0	0	0
6930	0	0	0
6689	0	0	0
Rad_Cap			
7472	50	50	0
5902	0	0	0
7090	110	108	2
7227	0	0	0
