# Supplementary material for: Effects of captopril against radiation injuries in the Göttingen minipig model of hematopoietic-acute radiation syndrome
Source: PLoS One. 2021 Aug 27;16(8):e0256208. doi: 10.1371/journal.pone.0256208 (PMC8396780; doi:10.1371/journal.pone.0256208)
Supplement: S4 File — This is a data file. (PDF) [file pone.0256208.s004.pdf]

EPO ELISA Gottingen minipigs

Software Version	2.00.18
Plate Number	Plate 1
Date	6/27/2018
Time	
Reader Type:	Synergy H1
Reader Serial Number:	264214
Reading Type	Reader

Procedure Details

Plate Type	96 WELL PLATE
Read	Absorbance Endpoint
	Full Plate
	Wavelengths: 450
	Read Speed: Normal, Delay: 100 msec, Measurements/Data Point: 8

Layout

	1	2	3	4	5	6	7	8	9	10	11	12	
A	10	582S+21d	80S+11d	A+4d	A+32d	A+25d	82R+18d	90R+11d	23RC+4d	23RC+32d	21RC+25d	5902RC+16	Well ID
B	5	582S+25d	80S+14d	A+7d	A-3d	A+28d	82R+21d	90R+14d	23RC+7d	21RC-3d	21RC+28d	5902RC+20	Well ID
C	0	582S+11d	80S+18d	A+11d	A+4d	A+32d	82R+25d	90R+18d	23RC+11d	21RC+4d	21RC+32d	7090RC+13	Well ID
D	582S-3d	582S+28d	80S+21d	A+14d	A+7d	82R-3d	82R+28d	90R+21d	23RC+14d	21RC+7d	6689R+16	7090RC+16	Well ID
E	582S+4d	582S+32d	80S+25d	A+18d	A+11d	82R+4d	82R+32d	90R+25d	23RC+18d	21RC+11d	6689R+20	7090RC+20	Well ID
F	582S+7d	80S-3d	80S+28d	A+21d	A+14d	82R+7d	90R-3d	90R+28d	23RC+21d	21RC+14d	7472RC+13	2164S+9	Well ID
G	582S+14d	80S+4d	80S+32d	A+25d	A+18d	82R+11d	90R+4d	90R+328d	23RC+25d	21RC+18d	7472RC+20	2164S+13	Well ID
H	582S+18d	80S+7d	A-3d	A+25d	A+21d	82R+14d	90R+7d	23RC-3d	23RC+28d	21RC+21d	5902RC+13	2164S+30	Well ID

Results s= Sham RC= Radiation + captopril R= radiation A = other treatment group

Actual Temperature: 24

	1	2	3	4	5	6	7	8	9	10	11	12	
A	2.288	0.082	0.068	0.074	0.072	0.086	0.143	0.093	0.057	0.065	0.16	0.092	450
B	1.33	0.065	0.077	0.082	0.061	0.059	0.13	0.068	0.067	0.073	0.171	0.075	450
C	0.085	0.115	0.066	0.063	0.061	0.081	0.086	0.079	0.096	0.064	0.199	0.086	450
D	0.083	0.083	0.066	0.094	0.065	0.071	0.068	0.079	0.059	0.063	0.076	0.436	450
E	0.073	0.07	0.069	0.088	0.078	0.065	0.06	0.07	0.063	0.121	0.083	0.668	450
F	0.067	0.069	0.061	0.08	0.075	0.141	0.09	0.081	0.079	0.087	0.065	0.316	450
G	0.071	0.07	0.061	0.131	0.086	0.161	0.112	0.069	0.063	0.091	0.286	0.319	450
H	0.088	0.064	0.065	0.106	0.1	0.078	0.119	0.068	0.069	0.12	0.097	0.109	450

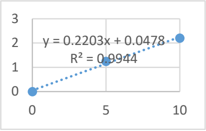

GM-CSF

Software Version 2.00.18

Plate Number Plate 1
Date 9/4/2018
Time ########
Reader Type: Synergy H1
Reader Serial Number: 264214
Reading Type Reader

Procedure Details

Plate Type 96 WELL PLATE
Read Absorbance Endpoint
Full Plate
Wavelengths: 550
Read Speed: Normal, Delay: 100 msec, Measurements/Data Point: 8
Read Absorbance Endpoint
Full Plate
Wavelengths: 450
Read Speed: Normal, Delay: 100 msec, Measurements/Data Point: 8

Layout

	1	2	3	4	5	6	7	8	9	10	11	12	
A	18000	S+16	S+9	A+2	A+35	A+23	R+16	R+9	R+2	R+6	RC+9	RC+2	Well ID
B	7200	S+20	S+13	A+6	A-5	A+30	R+20	R+13	R+6	R+9	RC+13	RC+6	Well ID
C	0	S+23	S+16	A+9	A+2	A+35	R+23	R+16	R+9	R+13	RC+16	RC+9	Well ID
D	S-5	S+30	S+20	A+13	A+6	R-5	R+30	R+20	R+13	R+16	RC+20	RC+13	Well ID
E	S+2	S+35	S+23	A+16	A+9	R+2	R+35	R+23	R+16	R+20	RC+23	RC+16	Well ID
F	S+6	S-5	S+30	A+20	A+13	R+6	R-5	R+30	R+20	RC-5	RC+30	RC+20	Well ID
G	S+9	S+2	RC+35	A+23	A+16	R+9	R+2	R+35	R-5	RC+2	RC+35	RC+23	Well ID
H	S+13	S+6	A-5	A+30	A+20	R+13	R+6	R-5	R+2	RC+6	RC-5	RC+30	Well ID

Results s= Sham RC= Radiation + captopril R= radiation A = other treatment group

Actual Temperature: 24
Actual Temperature: 24

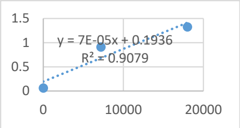

		1	2	3	4	5	6	7	8	9	10	11	12	
550	A	0.053	0.039	0.04	0.04	0.039	0.04	0.041	0.039	0.039	0.04	0.039	0.05	Read 1:550
450		1.377	0.07	0.056	0.164	0.069	0.065	0.075	0.061	0.067	0.128	0.06	0.071	Read 2:450
		1.324	0.031	0.016	0.124	0.03	0.025	0.034	0.022	0.028	0.088	0.021	0.021	Read 1:550
550	B	0.049	0.041	0.04	0.04	0.04	0.04	0.04	0.04	0.039	0.042	0.042	0.039	Read 2:450
450		0.955	0.073	0.063	0.131	0.073	0.066	0.078	0.076	0.067	0.177	0.072	0.086	Read 1:550
		0.906	0.032	0.023	0.091	0.033	0.026	0.038	0.036	0.028	0.135	0.03	0.047	Read 2:450
550	C	0.041	0.04	0.04	0.041	0.041	0.039	0.04	0.04	0.04	0.044	0.04	0.039	Read 1:550
450		0.1	0.066	0.065	0.099	0.06	0.056	0.077	0.076	0.074	0.111	0.074	0.069	Read 2:450
		0.059	0.026	0.025	0.058	0.019	0.017	0.037	0.036	0.034	0.067	0.034	0.03	Read 1:550
550	D	0.146	0.062	0.041	0.042	0.038	0.04	0.041	0.038	0.038	0.039	0.039	0.038	Read 2:450
450		0.182	0.102	0.074	0.09	0.055	0.063	0.062	0.08	0.075	0.067	0.072	0.085	Read 1:550
		0.036	0.04	0.033	0.048	0.017	0.023	0.021	0.042	0.037	0.028	0.033	0.047	Read 2:450
550	E	0.039	0.05	0.047	0.039	0.039	0.041	0.039	0.044	0.039	0.039	0.038	0.039	Read 1:550
450		0.069	0.088	0.08	0.073	0.062	0.061	0.064	0.089	0.069	0.061	0.059	0.069	Read 2:450
		0.03	0.038	0.033	0.034	0.023	0.02	0.025	0.045	0.03	0.022	0.021	0.03	Read 1:550
550	F	0.041	0.043	0.043	0.054	0.039	0.04	0.039	0.041	0.05	0.039	0.04	0.039	Read 2:450
450		0.068	0.075	0.071	0.073	0.072	0.163	0.089	0.081	0.092	0.064	0.056	0.069	
		0.027	0.032	0.028	0.019	0.033	0.123	0.05	0.04	0.042	0.025	0.016	0.03	
550	G	0.052	0.042	0.043	0.042	0.04	0.039	0.041	0.039	0.043	0.039	0.04	0.039	
450		0.082	0.215	0.075	0.065	0.073	0.068	0.063	0.083	0.093	0.053	0.053	0.066	
		0.03	0.173	0.032	0.023	0.033	0.029	0.022	0.044	0.05	0.014	0.013	0.027	
550	H	0.04	0.04	0.04	0.039	0.04	0.042	0.039	0.04	0.039	0.043	0.041	0.039	
450		0.066	0.105	0.07	0.067	0.067	0.081	0.061	0.066	0.063	0.06	0.064	0.068	
		0.026	0.065	0.03	0.028	0.027	0.039	0.022	0.026	0.024	0.017	0.023	0.029	

SAA ELISA

Software Version	2.00.18
Plate Number	Plate 2
Date	6/13/2018
Time	
Reader Type:	Synergy H1
Reader Serial Number:	264214
Reading Type	Reader

Procedure Details

Plate Type	96 WELL PLATE
Read	Absorbance Endpoint
	Full Plate
	Wavelengths: 450
	Read Speed: Normal, Delay: 100 msec, Measurements/Data Point: 8

Layout

	1	2	3	4	5	6	7	8	9	10	11	12	
A	1000	S+16d	S+9d	S+2d	S+35d								Well ID
B	500	S+20d	S+13d	S+6d									Well ID
C	0	S+23d	S+16d	S+9d									Well ID
D	S-5d	S+30d	S+20d	S+13d									Well ID
E	S+2d	S+35d	S+23d	S+16d									Well ID
F	S+6d	SC+35d	S+30d	S+20d									Well ID
G	S+9d	S+2d	S+35d	S+23d									Well ID
H	S+13d	S+6d	S-5d	S+30d									Well ID

Results

Actual Temperature: 26.6

	1	2	3	4	5	6	7	8	9	10	11	12	
A	2.697	0.267	0.367	0.106	0.096								450
B	1.79	0.25	0.749	0.111									450
C	0.144	0.918	0.376	0.179									450
D	1.441	0.569	0.109	2.216									450
E	0.224	0.274	0.39	0.305									450
F	0.131		0.766	0.648									450
G	0.123	0.347	0.42	0.1									450
H	0.74	0.69	0.214	0.17									450

Software Version	2.00.18
Plate Number	Plate 2
Date	6/5/2018
Time	
Reader Type:	Synergy H1
Reader Serial Number:	264214
Reading Type	Reader

Procedure Details

Plate Type	96 WELL PLATE
Read	Absorbance Endpoint
	Full Plate
	Wavelengths: 450
	Read Speed: Normal, Delay: 100 msec, Measurements/Data Point: 8

Layout

	1	2	3	4	5	6	7	8	9	10	11	12	
A	1000	S+2d	R-5d	R+2d	R+35d	R+23d	RC+20d	RC+23d	RC+16d	RC+9d	SPL81	SPL89	Well ID
B	500	S+16d	R+2d	R+6d	R-5d	R+30d	RC-5d	RC+30d	RC+20d	RC+13d	SPL82	SPL90	Well ID
C	0	S+30d	R+6d	R+9d	R+2d	R+35d	RC+2d	RC+35d	RC+23d	RC+16d	SPL83	SPL91	Well ID
D	S+6d		R+9d	R+13d	R+6d	RC-5d	RC+6d	RC-5d	RC+30d	RC+20d	SPL84	SPL92	Well ID
E	S+16d		R+13d	R+16d	R+9d	RC+2d	RC+9d	RC+2d	RC+35d	RC+23d	SPL85	SPL93	Well ID
F	S+23d		R+16d	R+20d	R+13d	RC+6d	RC+13d	RC+6d	RC-5d	RC+30d	SPL86	SPL94	Well ID
G	S+35d		R+20d	R+23d	R+16d	RC+9d	RC+16d	RC+9d	RC+2d	RC+35d	SPL87	SPL95	Well ID
H	S-5d	R+6d	R-5d	R+30d	R+20d	RC+13d	RC+20d	RC+13d	RC+6d	RC+16d	SPL88	SPL96	Well ID

Results

Actual Temperature: 23.3

	1	2	3	4	5	6	7	8	9	10	11	12	
A	2.625	0.942	0.847	0.232	0.286	0.426	1.083	0.183	0.407	0.198	0	0	450
B	1.428	0.701	0.379	0.203	0.611	0.234	0.167	0.262	0.51	0.204	0	0	450
C	0.198	0.418	0.283	0.659	0.334	0.231	0.313	0.169	0.972	0.298	0	0	450
D	0.216		0.272	0.454	0.752	0.172	0.192	0.159	0.41	0.252	0	0	450
E	0.365		1.065	0.748	0.231	0.226	0.193	0.269	0.21	0.23	0	0	450
F	0.37		1.656	0.493	0.223	0.172	0.2	0.17	0.52	0.262	0	0	450
G	0.776		1.782	0.336	0.72	0.209	0.297	0.19	0.287	0.198	0	0	450
H	0.559	0.167	0.569	0.328	0.517	0.918	0.341	0.609	0.193	1.072	0	0	450
