# Supplementary material for: Effects of captopril against radiation injuries in the Göttingen minipig model of hematopoietic-acute radiation syndrome
Source: PLoS One. 2021 Aug 27;16(8):e0256208. doi: 10.1371/journal.pone.0256208 (PMC8396780; doi:10.1371/journal.pone.0256208)
Supplement: S5 File — This is a data file. (PDF) [file pone.0256208.s005.pdf]

platelets	animal #	baseline	+2D	+6D	+7D	+9D	+13D
SHAM + V	6402	207.0	123.0	629.0		532.0	379.0
	3403	58.0	136.0	240.0		358.0	409.0
	2164	452.0	380.0	592.0		364.0	263.0
	3951	425.0	647.0	417.0		234.0	534.0
SHAM + CAP	4043	343.0	162.0	300.0		484.0	463.0
	3284	271.0	263.0	280.0		314.0	522.0
	6697	200.0	27.0	639.0		373.0	564.0
	6461	13.0	330.0	565.0		554.0	929.0
RAD + V	6496	156	483	653		244	17
	5091	357.0	130.0	323.0		102.0	14.0
	6930	40.0	474.0	430.0		118.0	22.0
	6689	215.0		439.0		98.0	10.0
RAD + CAP	7472	482.0	404.0	382.0		118.0	8.0
	5902	365.0	310.0	266.0		51.0	6.0
	7090	432.0	435.0	565.0		105.0	20.0
	7227	431.0	437.0	435.0		164.0	17.0
RAD + V	106 0406	219			197		
	106 0520	454			210		
	106 0121	644			446		
	106 0066	513			421		
RAD + CAP	106 0783	516			532		
	302 8852	578			561		
	302 8127	423			392		
	302 8941	541			413		
RAD + V	1104 896	703			60		
	1103 407	538			77		
	1104 381	461			212		
	1103 849	241			136		
RAD + CAP		635			146		
	1103 491	465			178		
	1104 462	457			188		
	1103 181	615			386		

+14D	+16D	+20D	+21D	+23D	+28D	+30D	+35D	
	421.0	682.0		562.0		720.0	360.0	
	364.0	693.0		397.0		510.0	317.0	
	814.0	352.0		614.0		767.0	520.0	
	516.0	714.0		709.0		708.0	780	
	544.0	619.0		530.0		405.0	419.0	
	424.0	487.0		330.0		444.0	375.0	
	484.0	695.0		366.0		723.0	19.0	
	628.0	612.0		705.0		809.0	472.0	
	18	20						* moribunc
	10.0	7.0						* moribunc
	7.0	19.0		24.0		13.0	18	
	7.0	14.0		11.0		26.0	60	
	7.0	8.0						* moribunc
	4.0	9.0		13.0		22.0	53	
	38.0	57.0		82.0		237.0	407	
	20.0	31.0		31.0		67.0	80	
4			13		47		136	
3								
12			39		64		43	
3			10		24		13	
4			10		38		72	
59			109		170		209	
9			33		59		123	
4			12		41		14	
7			8		6		5	
6			9		5		4	
3								
5			13		10		5	
4			3					
9			33		39		199	
11			13		20		5	
12			18		17		7	

- euthanized 21dpi
- euthanized 20dpi *not included - taken at death

- euthanized 20dpi

RBC	animal #	baseline	+2D	+6D	+7D	+9D	+13D
SHAM + V	6402	12.8	6.5	7.4		7.0	7.0
	3403	15.7	7.1	7.2		7.6	6.4
	2164	7.2	8.3	7.5		7.0	6.9
	3951	4.9	6.0	6.6		6.9	8.6
SHAM + CAP	4043	6.7	7.4	7.1		6.3	7.0
	3284	7.8	7.1	8.0		7.1	8.2
	6697	12.8	3.4	7.2		7.0	7.4
	6461	9.7	7.9	7.4		7.2	7.1
RAD + V	6496	15.47	7.69	7.47		6.73	7.21
	5091	6.52	6.84	7.98		7.59	7.18
	6930	12.69	5.6	6.6		6.0	6.0
	6689	8.55		7.4		6.8	7.4
RAD + CAP	7472	7.55	7.86	8.0		7.1	6.6
	5902	6.07	6.9	6.5		5.7	6.0
	7090	7.2	7.1	6.5		6.2	7.3
	7227	6.8	7.6	6.3		6.3	7.3
RAD + V	106 0406	6.21			6.67		
	106 0520	6.67			7.18		
	106 0121	6.17			6.78		
	106 0066	6			7.44		
RAD + CAP	106 0783	7.47			7.61		
	302 8852	8.41			8.07		
	302 8127	7.69			7.43		
	302 8941	6.96			6.88		
RAD + V	1104 896	7.65			10.34		
	1103 407	8.11			6		
	1104 381	8.13			9.08		
	1103 849	7.77			8.88		
RAD + CAP		8.53			7.62		
	1103 491	8.87			8.22		
	1104 462	7.06			6.52		
	1103 181	7.66			7.29		

+14D	+16D	+20D	+21D	+23D	+28D	+30D	+35D	
	6.9	6.6		7		6.6	6.3	
	6.4	7.1		7.39		6.9	6.9	
	8.7	6.8		7.65		7.0	6.6	
	7.8	8.4		8.13		7.7	7.04	
	6.5	6.7		6.82		7.2	6.62	
	7.5	7.4		7.58		7.2	6.36	
	6.8	6.6		7.34		7.4	5.82	
	6.9	7.0		7.7		7.0	7.18	
	4.68	4.76						* moribund
	5.89	1.05						* moribund
	5.7	5.4		4.74		4.78	4.17	
	6.1	6.4		6.18		5.69	5.11	
	5.1	1.9						* moribund
	5.5	4.9		5.25		4.9	4.72	
	7.0	7.2		8.2		7.39	6.41	
	6.8	6.4		5.8		5.3	5.37	
7.72			5.53		5.37		5.3	
6.74								
7.26			6.15		6.79		6.48	
6.81			5.55		5.56		5.97	
6.96			5.19		4.33		3.91	
7.26			8.18		7.06		5.97	
7.61			6.31		7.27		6.59	
8.86			5.45		6.3		6.3	
7.15			5.75		5.45		1.81	
6.17			6.35		6.59		4.94	
8.3								
7.4			7.26		7.37		5.34	
7.68			1.91					
8.45			8.13		8.95		6.4	
6.17			5.51		5.32		4.71	
6.61			6.56		6.43		5.72	

[illegible] - euthanized 21dpi	
[illegible] - euthanized 20dpi	*not included - taken at death
[illegible] - euthanized 20dpi	

RBC	animal #	baseline	+2D	+6D	+7D	+9D	+13D
SHAM + V	6402	68.7	34.6	40.0		38.9	38.5
	3403	80.5	37.1	36.9		39.7	33.9
	2164	34.2	38.6	34.8		33.5	32.5
	3951	25.9	34.7	37.8		38.6	46.9
SHAM + CAP	4043	32.5	37.2	36.1		32.3	37.1
	3284	38.0	33.8	38.5		34.6	39.1
	6697	63.7	16.4	35.7		35.5	36.7
	6461	49.4	38.9	37.0		36.1	37.0
RAD + V	6496	75.90	36.8	35.7		32.7	36
	5091	33.70	33.4	38.2		36.8	34.9
	6930	69.00	30.9	36		33.4	34
	6689	44.80		37.1		34.3	38.2
RAD + CAP	7472	35.90	37.3	38.6		33.7	31.6
	5902	32.70	37.2	35.4		31.2	33.9
	7090	37.4	36.0	32.9		30.8	37.2
	7227	34.9	39.9	33.3		34.1	39.4
RAD + V	106 0406	30.3			34.4		
	106 0520	33.9			39.9		
	106 0121	32.2			37		
	106 0066	29			39.8		
RAD + CAP	106 0783	31.7			33.2		
	302 8852	40.6			39.4		
	302 8127	35.8			35.1		
	302 8941	33			33.8		
RAD + V	1104 896	37.9			53.7		
	1103 407	39.4			30.1		
	1104 381	37.7			42.6		
	1103 849	38.9			44.7		
RAD + CAP		39.9			36.7		
	1103 491	41.2			39.7		
	1104 462	39.2			38.1		
	1103 181	35			34.1		

+14D	+16D	+20D	+21D	+23D	+28D	+30D	+35D	
	38.2	36.8		38.7		36.3	35.9	
	34.4	34.1		39.2		36.5	37.6	
	45.9	36.2		37.2		33.1	33.2	
	37.7	43.8		41		38.0	35.6	
	35.5	36.4		36.6		38.4	35.8	
	35.9	35.9		36.6		35.0	32.3	
	34.1	33.4		37.1		36.7	29.4	
	37.2	39.1		42.4		37.7	39.9	
	23.1	23.6						* moribunc
	29.6	5.7						* moribunc
	32.1	30.4		27		29.3	26.5	
	31.6	33.2		32.8		31.3	29.9	
	25.0	10.0						* moribunc
	30.7	28.2		31.2		31.3	31.7	
	36.0	38.0		42.7		38.3	33.8	
	36.4	36.1		33.7		31.9	32.3	
41.3			29.6		30.4		31	
38.1								
42.3			36.5		41.3		39.7	
38.1			32.3		34.7		39.8	
31.7			23.9		20.9		21.1	
36.4			42.9		37.4		32.2	
37.4			31		37.7		34.8	
46.1			27.7		35.3		37.7	
37.5			31.3		31.5		10.9	
32.8			35.8		38.4		28.9	
39.2								
37.9			38.1		39.3		28.4	
37.9			9.4					
42.3			42.1		46.7		33	
37.2			34.9		35.6		31.8	
32.7			33.6		35.2		30.7	

- euthanized 21dpi
- euthanized 20dpi *not included - taken at death

- euthanized 20dpi

RBC

	animal #	baseline	+2D	+6D	+7D	+9D	+13D
SHAM + V	6402	5.3	3.9	5.6		4.0	4.5
	3403	5.1	3.9	3.1		4.1	4.1
	2164	2.4	4.5	3.8		3.9	3.8
	3951	0.7	6.8	6.9		6.9	4.8
SHAM + CAP	4043	1.7	4.9	4.8		6.1	2.9
	3284	6.2	5.9	3.8		4.5	5.8
	6697	2.7	0.8	3.4		3.4	4.4
	6461	1.9	5.3	6.3		5.2	4.7
RAD + V	6496	4.05	3.17	1.09		1.34	0.31
	5091	3.40	2.09	5.49		3.25	0.39
	6930	1.76	2.7	2.31		2.75	1.1
	6689	3.17		1.76		0.9	0.29
RAD + CAP	7472	5.48	3.3	1.0		1.4	0.2
	5902	7.40	2.9	1.2		1.3	0.1
	7090	4.1	5.09	1.8		1.1	0.4
	7227	2.3	5.5	1.5		1.2	1.1
RAD + V	106 0406	3.7			2.09		
	106 0520	6.03			3.57		
	106 0121	4.39			0.9		
	106 0066	3.44			1.38		
RAD + CAP	106 0783	6.76			1.27		
	302 8852	4.44			0.88		
	302 8127	3			1.87		
	302 8941	2.8			2.02		
RAD + V	1104 896	3.21			2.23		
	1103 407	3.9			1.17		
	1104 381	2.42			1.68		
	1103 849	4.99			3.16		
RAD + CAP		3.71			1.98		
	1103 491	4.89			2.16		
	1104 462	3.04			1.62		
	1103 181	7.2			3.2		

+14D	+16D	+20D	+21D	+23D	+28D	+30D	+35D	
	3.6	4.6		5.39		4.9	5.82	
	3.7	8.8		3.69		4.5	4.07	
	6.1	4.3		5.87		4.5	6.52	
	4.5	5.2		12.4		9.4	6.82	
	3.5	6.1		5.46		7.4	2.79	
	5.4	9.2		7.46		9.2	6.14	
	3.7	6.7		5.9		7.0	6.34	
	6.7	3.8		6.45		6.6	5.37	
	0.11	0.31						* moribunc
	0.02	0.01						* moribunc
	0.32	0.91		1.59		1.63	1.83	
	0.09	0.3		0.47		0.63	1.34	
	0.1	0.3						* moribunc
	0.1	0.3		0.38		0.78	1.29	
	0.4	0.9		2.37		2.88	4.58	
	0.4	0.5		1.01		1.1	1.13	
0.56			0.3		0.48		1.14	
0.16								
0.48			0.64		1.13		1.24	
0.61			0.28		0.47		0.95	
0.44			0.35		0.5		1.34	
0.8			0.6		1.08		2.02	
0.32			0.29		0.69		1.29	
0.63			0.27		0.37		0.86	
0.47			0.17		0.34		0.01	
0.54			0.16		0.47		0.49	
0.23								
0.91			0.37		0.51		0.36	
0.41			0.04					
1.25			0.89		1.45		1.45	
0.47			0.37		0.92		0.76	
0.42			0.39		0.54		0.54	

- euthanized 21dpi

- euthanized 20dpi *not included - taken at death

- euthanized 20dpi

RBC	animal #	baseline	+2D	+6D	+7D	+9D	+13D
SHAM + V	6402	5.7	1.9	2.9		2.7	1.9
	3403	5.3	3.2	3.3		4.3	3.8
	2164	2.8	3.1	3.2		3.0	0.7
	3951	9.9	3.7	3.6		3.1	3.1
SHAM + CAP	4043	2.2	3.4	3.8		2.6	3.0
	3284	5.0	3.4	3.6		3.2	3.6
	6697	3.3	0.6	2.2		1.7	2.1
	6461	1.3	1.8	4.1		3.9	2.1
RAD + V	6496	5.30	0.14	1.23		1.41	1.08
	5091	5.43	0.61	1.37		1.56	0.95
	6930	4.69	0.92	1.37		1.52	1.15
	6689	3.02		0.91		0.59	0.69
RAD + CAP	7472	3.86	1.16	1.14		1.09	0.71
	5902	6.01	1.0	0.8		0.8	0.7
	7090	3.4	0.9	1.0		0.5	1.1
	7227	3.9	0.58	0.5		1.0	1.1
RAD + V	106 0406	2.43			0.5		
	106 0520	4.36			0.83		
	106 0121	4.44			1.2		
	106 0066	3.44			0.75		
RAD + CAP	106 0783	2.95			0.84		
	302 8852	3.44			0.61		
	302 8127	3.43			0.53		
	302 8941	5.01			1.58		
RAD + V	1104 896	2.34			0.42		
	1103 407	1.27			0.2		
	1104 381	3.07			0.69		
	1103 849	4.99			1.15		
RAD + CAP		3.57			0.57		
	1103 491	2.77			0.71		
	1104 462	3.18			0.68		
	1103 181	2.44			0.86		

+14D	+16D	+20D	+21D	+23D	+28D	+30D	+35D	
	1.7	2.3		2.75		2.5	2.13	
	3.7	3.7		3.39		3.7	3.47	
	5.0	3.6		4.82		3.4	3.31	
	3.9	4.4		4.16		3.3	4.91	
	3.0	1.8		2.99		1.7	2.24	
	3.7	3.9		3.58		4.1	2.97	
	1.1	1.9		1.86		2.4	1.66	
	3.5	2.8		3.26		3.6	2.47	
	0.95	0.91						* moribunc
	1.07	0.08						* moribunc
	1.02	1.46		1.82		2.21	2.21	
	0.57	0.9		1.26		1.27	1.18	
	1.08	1.0						* moribunc
	0.9	1.0		1.62		1.69	1.31	
	1.5	2.0		2.3		2.77	1.79	
	0.7	1.1		1.11		1.37	1.35	
1.63			1.4		1.46		1.79	
0.9								
1.2			1.62		1.95		2.13	
1			0.82		1.3		1.74	
0.8			1.01		1.37		1.95	
0.7			1.11		1.19		1.23	
0.71			0.96		1.32		1.56	
1.9			1.95		2.33		4.02	
0.73			0.51		1.06		0.03	
0.37			0.42		0.89		0.81	
0.73								
1.19			1.4		1.71		1.66	
1.16			0.66					
0.84			1.33		1.66		1.61	
0.81			0.94		1.11		1.46	
0.73			0.71		1.17		0.87	

- euthanized 21dpi
- euthanized 20dpi *not included - taken at death

- euthanized 20dpi

This sheet intentionally left blank

RBC	animal #	baseline	+2D	+6D	+9D	+13D	+16D
SHAM + V	6402		330.0	166.0	139.0	138.0	133.0
	3403	151.0	188.0	177.0	154.0	141.0	135.0
	2164	140.0	222.0	142.0	162.0	81.0	136.0
	3951	107.0	81.0	136.0	133.0	128.0	99.0
SHAM + CAP	4043	140.0	132.0	139.0	153.0	171.0	130.0
	3284	122.0	114.0	114.0	118.0	141.0	131.0
	6697	119.0	138.0	135.0	159.0	182.0	184.0
	6461	144.0	125.0	119.0	140.0	149.0	172.0
RAD + V	6496	148	132	149	153	129	94
	5091	140.00	113	126	152	123	76
	6930	117.00	191	160	143	120	98
	6689	131.00	156	112	109	127	82
RAD + CAP	7472	135.00	136	132	133	96	78
	5902	416.00	137	244	296	365	259
	7090	137.00	140.0	139.0	133.0	104.0	94.0
	7227	169.0	180.0	169.0	151.0	171.0	167.0

+20D	+23D	+30D	+35D		
127.0	105	106.0	104		
108.0	144	121.0	139		
154.0	100	84.0	126		
101.0	103	90.0	93		
144.0	128	142.0	118		
91.0	131	123.0	105		
145.0	128	122.0	90		
145.0	159	162.0	151		
79				* moribund - euthanized 21dpi	
96				* moribund - euthanized 20dpi	*not included - taken a
79	79	84	77		
85.0	96	104	100		
43.0				* moribund - euthanized 20dpi	
182.0	281	254	222		
84.0	81	78	91		
147.0	186	160	155		

ıt death

RBC	animal #	baseline	+2D	+6D	+9D	+13D	+16D
SHAM + V	6402		1	0.9	0.8	0.8	0.8
	3403	0.9	0.7	0.9	0.7	0.8	0.9
	2164	1.1	1.1	1	1.1	1.2	0.9
	3951	0.8	0.5	0.5	0.6	0.9	1.1
SHAM + CAP	4043	0.9	0.9	0.9	0.9	1.1	1.2
	3284	1.2	0.8	1.1	0.9	1.4	1.2
	6697	1.0	0.8	0.9	0.9	1	1
	6461	1.2	0.7	0.8	0.7	0.8	0.9
RAD + V	6496	0.83	0.8	0.8	0.8	1	0.9
	5091	1.07	0.9	1	1.1	1.2	1.2
	6930	0.76	1.1	0.6	0.8	0.8	0.8
	6689	1.03	0.9	1	0.9	1	0.9
RAD + CAP	7472	0.89	0.7	0.8	0.8	0.8	0.8
	5902	0.83	1	0.7	0.9	0.8	0.8
	7090	1.01	0.8	0.9	1	1.1	1.2
	7227	0.96	0.8	0.8	1	0.9	0.9

+20D	+23D	+30D	+35D		
0.9	0.7	0.8	0.8		
1	0.7	0.9	0.9		
0.7	1.2	1	1.1		
0.8	0.7	0.6			
1	0.9	0.9	1		
1.2	1.2	1	1.1		
0.8	0.9	1	0.8		
0.8	0.8	0.8	1		
0.9				* moribund - euthanized 21dpi	
14				* moribund - euthanized 20dpi	*not included - taken a
0.6	0.6	0.7	0.8		
0.9	0.8	0.8	0.7		
0.9				* moribund - euthanized 20dpi	
0.8	0.7	0.8	0.9		
1.1	1.1	0.9	1.1		
0.9	0.8	0.7	0.9		

ıt death

RBC	animal #	baseline	+2D	+6D	+9D	+13D	+16D
SHAM + V	6402		9	5	4	5	8.0
	3403	10.0	12	12	7	7	9.0
	2164	92.0	13	4	7	12	7.0
	3951	12.0	9	10	8	12	8.0
SHAM + CAP	4043	8.0	12	6	7	5	10.0
	3284	15.0	10	8	7	11	10.0
	6697	8.0	11	4	7	4	8.0
	6461	23.0	9	7	8	5	5.0
RAD + V	6496	5	9	8	11	11	10
	5091	10	11	8	11	12	10
	6930	5	12	6	6	8	7
	6689	105	14	9	9	10	10
RAD + CAP	7472	4	6	3	4	6	10.0
	5902	73	13	6	11	7	8.0
	7090	5	4	4	7	8	9.0
	7227	8.0	7	7	12	9	6.0

+20D	+23D	+30D	+35D		
7	6	7	9		
12	10	8	11		
7	15	9	11		
12	10	10			
5	4	8	8		
10	10	9	11		
4	10	9	8		
5	7	7	9		
14				* moribund - euthanized 21dpi	
48				* moribund - euthanized 20dpi	*not included - taken a
10	8	8	12		
11	11	12	11		
27				* moribund - euthanized 20dpi	
8	5	5	7		
7	10	7	10		
8	10	6	9		

ıt death

RBC	animal #	baseline	+2D	+6D	+9D	+13D	+16D
SHAM + V	6402		150.0	78.0	70	85.0	94
	3403	84.0	95.0	84.0	90	98.0	109
	2164	8.0	113.0	89.0	83	99.0	117
	3951	123.0	99.0	87.0	81	138.0	117
SHAM + CAP	4043	90.0	88.0	70.0	78	80.0	85
	3284	77.0	89.0	68.0	68	87.0	85
	6697	81.0	103.0	83.0	73	82.0	110
	6461	104.0	86.0	85.0	73	93.0	90
RAD + V	6496	88	63	81		116	53
	5091	83	80	81	86	106	90
	6930	94	115	96	90	125	91
	6689	7	125	94	97	110.0	97
RAD + CAP	7472	101	87	94	91	108.0	96
	5902	6	96	90	88	118.0	113
	7090	116	97	78	90	104.0	95
	7227	92.0	87.0	81.0	86	97.0	95

+20D	+23D	+30D	+35D		
85	78	80	71		
84	94	102	99		
90	96	103	95		
107	92	84			
77	91	84	75		
85	83	92	49		
80	86	86	66		
82	99	85	88		
65				* moribund - euthanized 21dpi	
74				* moribund - euthanized 20dpi	*not included - taken a
114	102.0	109	84		
117	119.0	114	119		
86				* moribund - euthanized 20dpi	
89	121	115	128		
101	111	97	87		
87	96	97	103		

ıt death

RBC	animal #	baseline	+2D	+6D	+9D	+13D	+16D
SHAM + V	6402		78	46.0	48	46	65
	3403	27.0	62	91.0	68	60	79
	2164	47.0	92	71.0	95	71	110
	3951	19.0	49	51.0	50	159	74
SHAM + CAP	4043	55.0	118	85.0	85	68	108
	3284	72.0	107	71.0	78	96	111
	6697	46.0	94	116.0	120	100	111
	6461	39.0	78	60.0	65	64	81
RAD + V	6496	51	112	94	88	74	60
	5091	58	112	101	100	84	74
	6930	25	71	48	61	60	59
	6689	60	64	92	77	82	87
RAD + CAP	7472	34	70	57	64	61	77
	5902	30	121	60	63	67	58
	7090	40	74	61	73	69	76
	7227	29.0	65	50.0	63	81	83

+20D	+23D	+30D	+35D		
76	90	85	80		
92	87	77	89		
83	88	75	96		
147	110	67	86		
100	92	80	79		
112	112	95	96		
125	117	89	90		
83	84	93	98		
63				* moribund - euthanized 21dpi	
52				* moribund - euthanized 20dpi	*not included - taken a
53	54	56	66		
100	102	87	86		
58				* moribund - euthanized 20dpi	
77	89	73	90		
86	93	83	121		
96	95	75	86		

ıt death
